# Optimized Properties and Synthesis of Photoactivatable Diazoketorhodamines Facilitate and Enhance High-Throughput Single-Molecule Tracking

**DOI:** 10.1021/acs.joc.4c00718

**Published:** 2024-06-05

**Authors:** Nicholas W. Pino, Anne R. Sizemore, Leah Cleary, Helen Liu, David T. McSwiggen, Dan Song, Hilary P. Beck, Kylie Cheng, Miki Hardy, Jessica Hsiung, Yangzhong Tang, Rajender Anugula, Santhosh Lakshman, Ravi K. Merneedi, Pradipta Sinha

**Affiliations:** † Eikon Therapeutics Inc., Hayward, California 94545, United States; ‡ Aragen Life Sciences Ltd., Bengaluru 562106, India

## Abstract

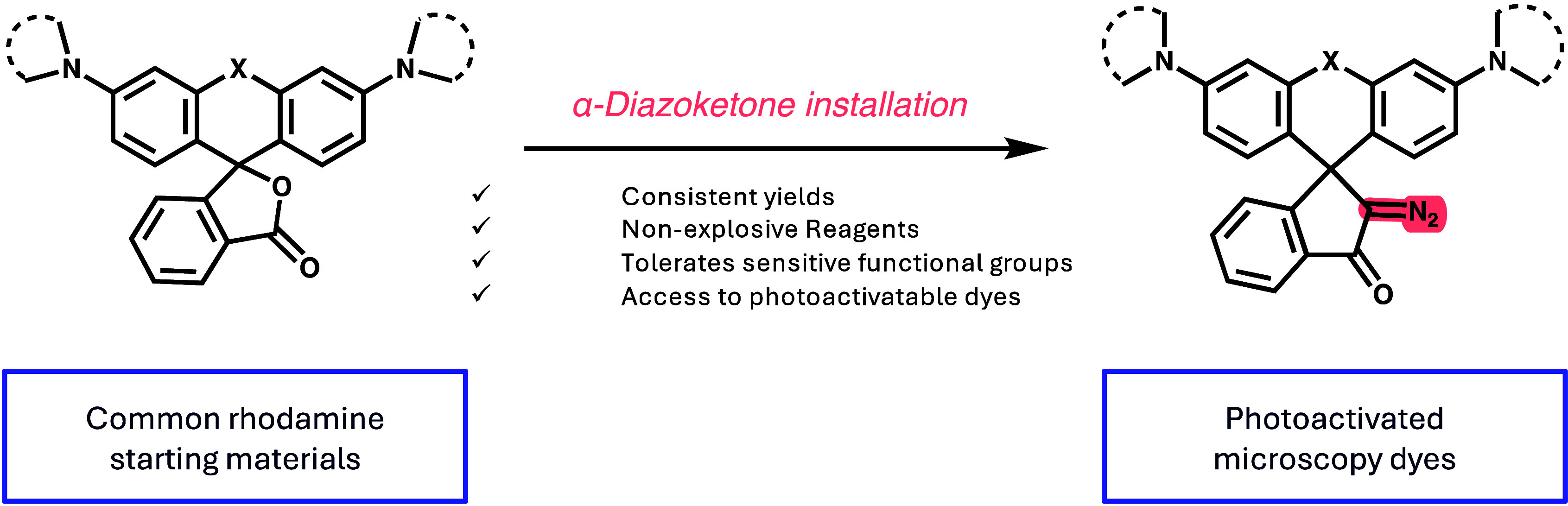

Photoactivatable (PA) rhodamine dyes are widely used in single-molecule tracking (SMT) and a variety of other fluorescence-based imaging modalities. One of the most commonly employed scaffolds uses a diazoketone to lock the rhodamine in the nonfluorescent closed form, which can be activated with 405 nm light. However, poor properties of previously reported dyes require significant washing, which can be resource- and cost-intensive, especially when performing microscopy in a large scale and high-throughput fashion. Here, we report improved diazoketorhodamines that perform exceptionally well in single-molecule tracking microscopy. We also report on the optimization of an improved synthetic method for further iteration and tailoring of diazoketorhodamines to the requirements of a specific user.

## Introduction

**Scheme 1:**
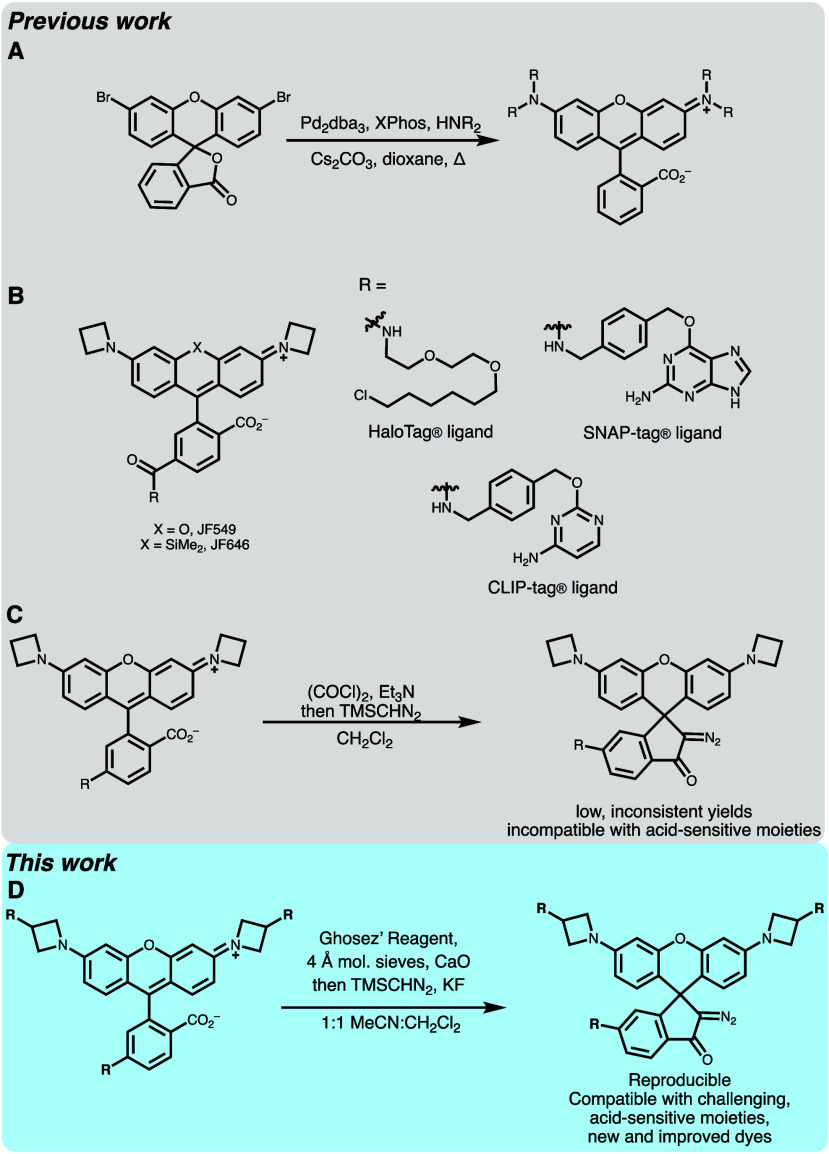
(A) Synthesis of Rhodamines via Buchwald–Hartwig Coupling to Rapidly Access Rhodamines from Bromofluorans; (B) Structures of Canonical JaneliaFluor Dyes with Varying Substitutions To Tune Wavelength (X) and Protein Tag (R); (C) Previous Method Reported for the Synthesis of Diazoketone-Caged Rhodamines; (D) Improved Method from This Work To Synthesize Diazoketone-Caged Rhodamines

Rhodamine dyes have been used extensively as fluorescent reporters in a wide variety of biological imaging applications due to their robust photophysical properties and high quantum yields.^1−3^ Pioneering work by Lavis and co-workers improved upon classical syntheses^1^ of these valuable small molecule dyes by employing a versatile Buchwald–Hartwig coupling strategy to install different *N*-alkyl substituents onto the rhodamine core, with azetidine-based groups providing a notable increase in quantum yield (Scheme 1A).^4,5^ Expanding this methodology to silicon- and carbo-rhodamine congeners afforded a suite of JaneliaFluor dyes with excitations across the visible spectrum that could be fine-tuned by changing the azetidine substitution pattern.^6−8^

**Figure 1:**
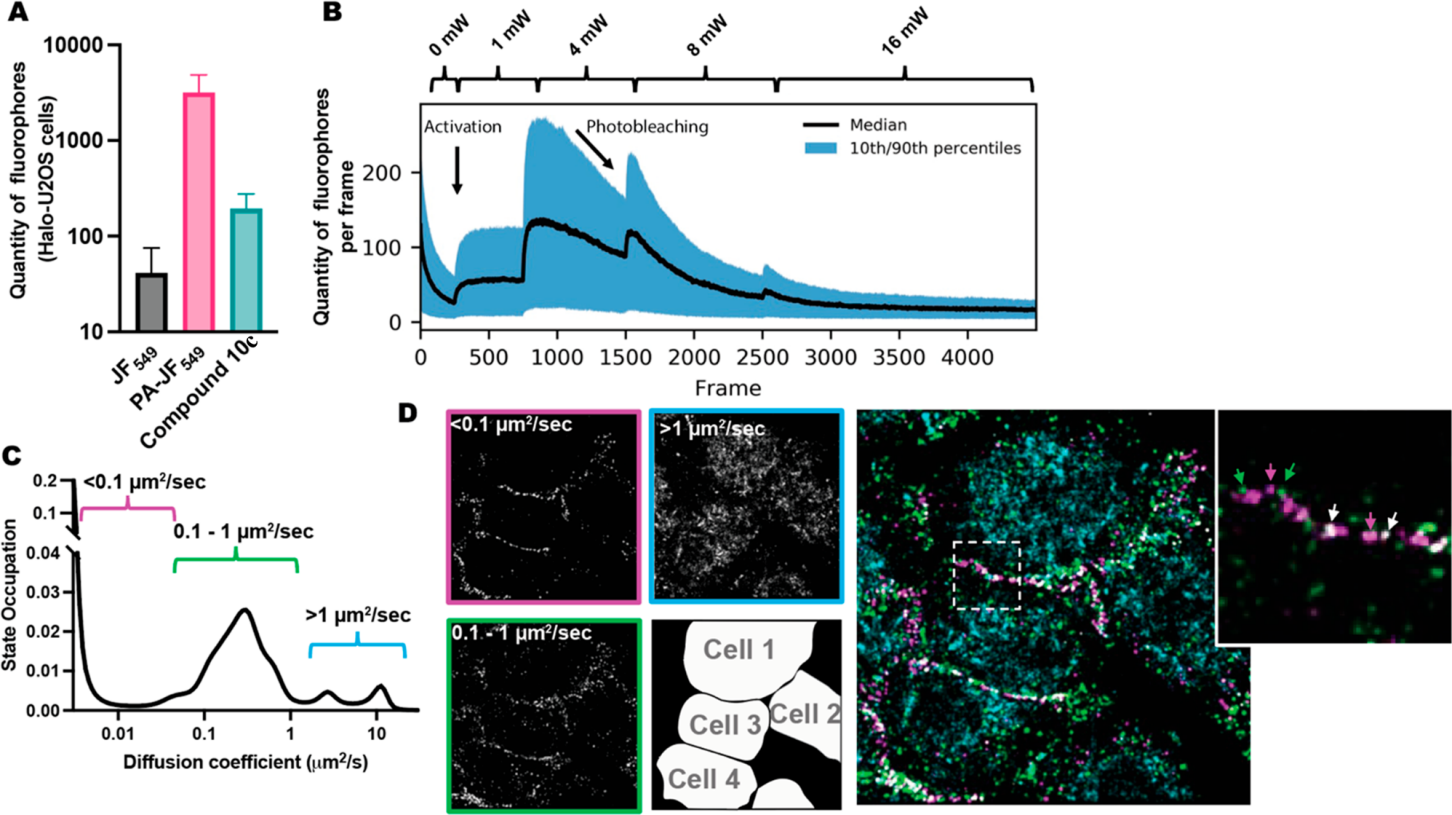
(A) Number of spots detected in HaloTag-negative U2OS cells with JF549-HaloTag (gray), photoactivatable JF549-HaloTag (pink), and **10c** (green) depicting that **10c** mitigates nonspecific labeling compared to photoactivatable JF549-HaloTag. (B) Time course over 4000 frames depicting photoactivation events of **10c** at 1 mW, 4 mW, and 8 mW and subsequent loss of signal due to photobleaching. (C) Graphical representation of occurrence of **10c** tagged to β-catenin-HaloTag fusion protein across diffusion coefficients and marked as populations that are slow (<0.1 μm^2^/s, magenta), moderate (0.1–1 μm^2^/s, green), and fast (>1 μm^2^/s, blue) diffusing populations. (D) (Right) Field of view containing 4 cells with populations described in C coded by color. Image indicates that β-catenin exists in slow to moderate diffusion rate populations at the focal adhesion regions compared with a fast population inside the cell. Cutout zooms in on two distinct slow and moderate populations at the focal adhesion region between cell 1 and cell 3. (Left) 3 channels depicted separately clearly indicating the location of each population and a cell localization map.

Advances in the rational design of rhodamine dyes have led to their application in live-cell super-resolution microscopy^9,10^ and single-molecule tracking experiments.^6,11^ Many of these studies require the attachment of a protein-specific ligand to the rhodamine core (Scheme 1B), such as HaloTag, SNAP-tag, and CLIP-tag groups, that can covalently bind, or “tag,” a genetically modified protein of interest.^9,10,12,13^ For many, JF549 and JF646 with one of the listed tags have become workhorse dyes for microscopy applications across the field (Scheme 1B). The development of α-diazoketone-containing caged dyes which are nonfluorescent until activation by an external light source has enabled photoactivated localization microscopy (PALM), a super-resolution microscopy technique that has been employed widely for a highly accurate study of biological processes.^14−17^ As the name suggests, PALM and single particle tracking Photoactivation Localization Microscopy (sptPALM) make use of the photoactivatable nature of fluorophores to achieve the sparsity needed for super-resolution reconstruction. We recently reported a high-throughput single-molecule tracking assay that operates at a scale of thousands of wells per day.^18^ Using first generation photoactivatable JaneliaFluor dyes, we found that the photoactivatable diazoketone of JF549-HaloTag shows >1000-fold more nonspecific single-molecule localizations when incubating and washing in U2OS cells compared to the uncaged JF549-HaloTag (Figure 1A). To prevent nonspecific staining, we required an unacceptably high volume and quantity of wash steps, which was incompatible with the scale of our methodology.

Inspired to improve the nonspecific background labeling observed using JF549-HaloTag derived diazoketones, we sought to modify these dyes to improve their properties. As reported previously, the diazoketone can be introduced at a late stage by treating the parent rhodamine dye with oxalyl chloride, followed by diazomethane or TMS-diazomethane (Scheme 1C).^14,15^ In our hands, freshly prepared diazomethane provided more reliable yields; however, it is hazardous and provides insufficiently improved yields and reproducibility to justify its use. Additionally, we found that the reported method was incompatible with acid-sensitive functional groups. Overall, these synthetic challenges broached a need for an improved method for the synthesis of diazoketones on rhodamine dyes. In the interest of developing a broadly applicable method, we set out to improve the activation strategy of the carboxylate for the addition of a diazomethane synthon.

## Results and Discussion

### Optimization of Novel Dye Synthesis

**Scheme 2:**
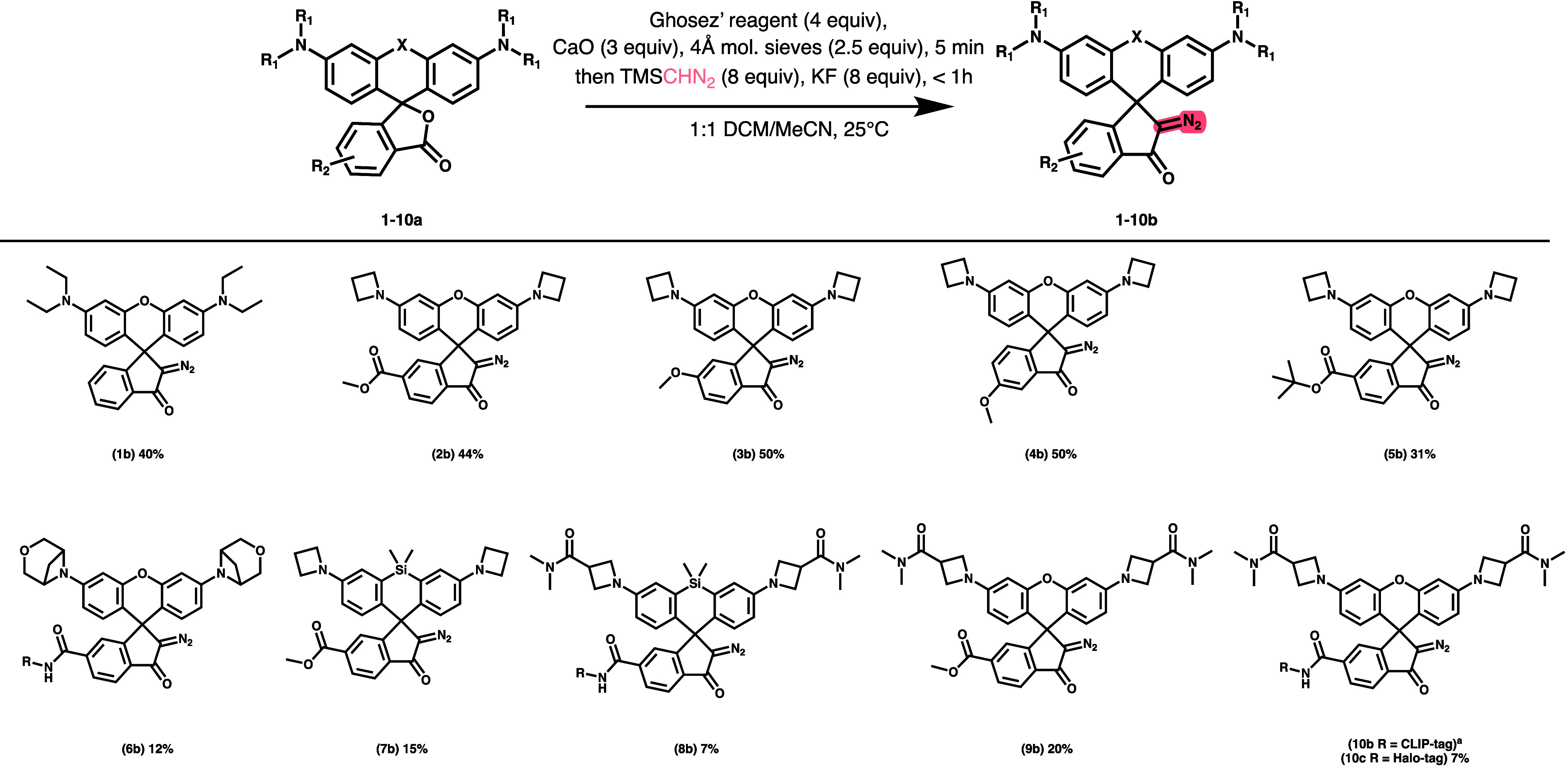
Scope of Photoactivatable Rhodamines Synthesized by Improved Diazoketone Synthesis Method (For **10c**, R = HaloTag Ligand (See Scheme 1)) **10b** was accessed via saponification and coupling of CLIP-tag to **9b**, not directly from the corresponding rhodamine lactone.

We began by screening various reagents that could activate the carboxylate sufficiently to react with TMSCHN_2_, a safer alternative to diazomethane. Starting with previously reported conditions on JF549-COOMe (**2a**, see Scheme 2),^14,15^ we observed formation of the acid chloride upon treatment of the free rhodamine with oxalyl chloride as expected. However, we also observed multiple chlorinated side products resulting from an acid-mediated ring opening of one or both azetidine rings to give 3-chloropropanamine side products. This undesired reaction was most likely promoted by residual HCl present in oxalyl chloride.

In an effort to minimize the formation of chlorinated byproducts, we explored anhydride formation as an activating strategy. In contrast to previous reports on different substrates, no reaction was observed when JF549-COOMe (**2a**) was treated with Boc-anhydride or ethyl chloroformate (Table S1, entries 3–4).^19^ It is hypothesized that this lack of reactivity results from the highly sterically hindered environment. Isobutyl chloroformate did form the expected mixed anhydride, as observed by LC-MS reaction monitoring; however, treatment with TMSCHN_2_ gave no reaction (Table S1, entry 5). This result indicated to us that anhydrides are not sufficiently activating for this application.

**Scheme 3:**
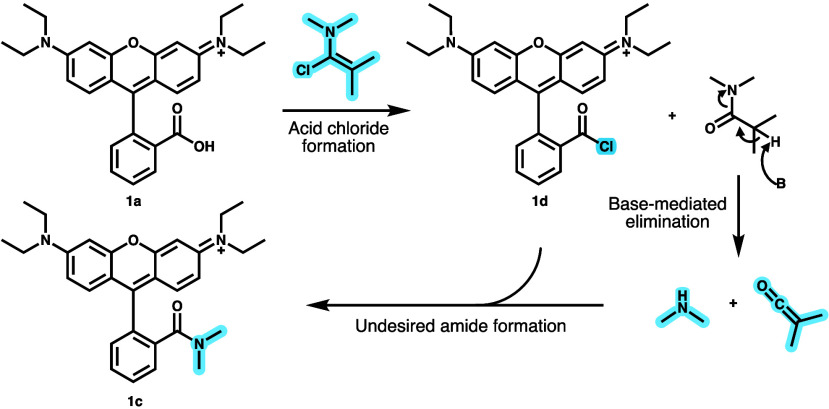
Putative Source of Dimethylamine after Acyl Chloride Formation with Ghosez’s Reagent and Subsequent Undesired Amide Formation to **1c**

Similarly, despite previous reports,^19^ common carboxylate activating reagents including HATU, PyBOP, PyClOP, and PyBrOP formed the activated intermediate by LC-MS, but all intermediates were unreactive to TMSCHN_2_ (Table S1, entries 6–9). Given the general lack of reactivity without acid chloride formation, we elected to revisit acid chloride formation with more gentle conditions. 1-Chloro-*N,N*,2-trimethyl-1-propenylamine, also known as Ghosez’s reagent (Scheme 3),^20,21^ rapidly and cleanly converted **1a** to the acid chloride (**1e**), which upon treatment with TMSCHN_2_ provided the desired α-diazoketone (**1b**) in modest yields.

When applied to an azetidine-containing substrate (**2a**) Ghosez’s reagent resulted in a notable reduction in the formation of azetidine ring-opened side products, likely due to the reduced amount of free HCl in the system. Nevertheless, the reaction suffered from poor yields and inconsistent product distributions. One hypothesis was that the starting material or acid chloride intermediate may not be fully soluble in dichloromethane. An important consideration in selecting a solvent in this reaction is stabilization of the open form of the compound, such that the carboxylate is available to react with Ghosez’s reagent. Prior reports have shown that moderately polar environments stabilize the charge buildup of the zwitterionic open form of the dye;^3^ however, sufficiently nonpolar solvent is required for solubility at synthetically relevant concentrations. A screen of polar aprotic solvents was conducted (Table S2) and unexpectedly yielded no single-solvent system with substantially improved reactivity. Noticing that the most promising results were seen in dichloromethane and acetonitrile, we screened a mixture of the two solvents and discovered that the rhodamines were soluble in a mixture of dichloromethane and acetonitrile (Table S2, entries 1–3). Upon addition of Ghosez’s reagent to this solution, the reaction turned a deep pink color, indicating that the open form was dominant in the solution. After treatment of an aliquot of reaction mixture with methylamine, rapid conversion of the acyl chloride to the colorless methyl lactam was observed, providing a convenient strategy to determine acyl chloride formation with the naked eye.

While this solvent system improved the reaction, at times the conversion to the acid chloride would remain incomplete even after successive additions of Ghosez’s reagent. Despite using oven-dried glassware and commercially available extra dry solvents, we reasoned that the reaction was particularly sensitive to trace amounts of water, causing the reaction to stall. To remove adventitious water, we allowed the reaction mixture to stir with 4 Å molecular sieves for 10 min prior to adding Ghosez’s reagent, which significantly improved reproducibility of the reaction over multiple batches and substrates (Table 1, entries 1–5).

**Table 1:**
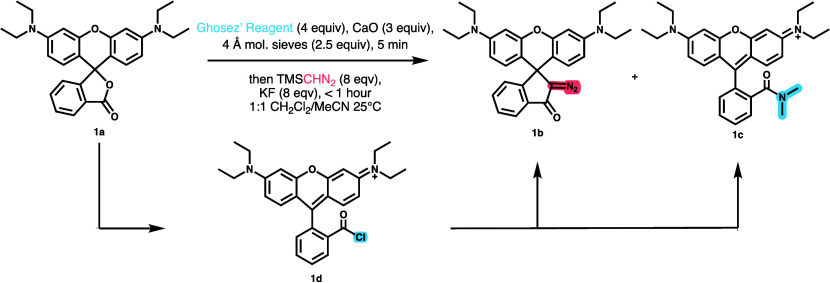
Optimization of Acid Chloride and Subsequent Diazoketone Synthesis

Entry^a^	Variation from standard conditions	**1a** (%)^b^	**1b** (%)^b^	**1c** (%)^b^	**1d** (%)^b^^,^^c^
1	None	0	100	0	0
2	4 equiv TMSCHN_2_, 4 equiv KF	0	72	28	0
3	2 equiv TMSCHN_2_, 2 equiv KF	0	34	54	12
4	2 equiv TMSCHN_2_, 2 equiv KI	12	50	31	7
5	2 equiv TMSCHN_2_, no KF	0	63	26	3
6	2 equiv TMSCHN_2_, 4 equiv KI, no 4 Å mol. sieves	55	23	20	3
7	2 equiv TMSCHN_2_, 4 equiv KBr, no 4 Å mol. sieves	45	21	29	5
8	2 equiv TMSCHN_2_, no KF, no 4 Å mol. sieves	55	10	33	3
9	2 equiv TMSCHN_2_, no KF, no 4 Å mol. sieves, no CaO	28	16	48	8
10	2 equiv TMSCHN_2_, no KF, no 4 Å mol. sieves, 4 equiv DIPEA	67	0	0	33

a Reactions run on a 0.1 mmol scale.

b Product distribution determined by LC-MS using percent of total area under the curve.

c Percent **1d** evaluated as the corresponding methyl amide after quenching the reaction mixture with methylamine.

Confident that the acid chloride could be formed reliably and reproducibly, we optimized the installation of the diazoketone with TMSCHN_2_. Owing to its reduced nucleophilicity compared to diazomethane, we anticipated that some optimization would be necessary to facilitate use of the safer, more convenient reagent.^22^ Using Hünig’s base, we observed smoking upon addition, indicating rapid formation of HCl (Table 1, entry 10). With this condition, we also observed formation of the dimethyl amide (**1c**) forming at the carboxylic acid of the rhodamine (Table 1 and Scheme 3). We hypothesize that Hünig’s base caused decomposition of the Ghosez reagent byproduct, liberating an equivalent of dimethylamine which then attacked the acid chloride to form the dimethyl amide (Scheme 3). A prior report from De Kimpe and co-workers inspired us to address this issue with a weaker base, CaO, to prevent dimethylamine formation.^23^ To our delight, CaO worked with our conditions for cyclic diazoketone synthesis (Table 1, entries 9–10). Despite the reduction in dimethyl amide formation, we observed that the diazoketone installation stalled before all of the acid chloride was consumed. We hypothesized that further activating the acid chloride or activating the TMSCHN_2_ might push the reaction to completion. Inspired by others, we first tried using KBr and KI to form the transient acid bromide and iodide respectively (Table 1, entries 4, 6–7).^24^ In each case, only a modest improvement was observed. Conversely, 1 equiv of KF per equivalent of TMSCHN_2_ assisted diazoketone installation, ultimately driving the reaction completion with increased equivalents of TMSCHN_2_ based on LC-MS reaction monitoring (Table 1, entries 1–3). Owing to the high affinity of fluoride to silicon, we propose that the fluoride from KF can deprotect TMSCHN_2_ to restore the nucleophilicity of unprotected diazomethane.^22^ With these conditions, the reaction gave consistent yields on scales ranging from 0.1 mmol (50 mg) to 2.25 mmol (1.0 g) on our test substrate, **1a** (see Supporting Information). Consequently, we took these conditions into account to adjudicate the reaction further. To assess scope, we applied the method to a variety of azetidine-containing rhodamines with electron donating or withdrawing groups on the bottom aryl ring (**2**–**10**). In all cases, we observed consistent yields. We were particularly interested in the successful synthesis of strained azetidine-containing compounds such as **6b**, which we were unable to synthesize using previously reported methods due to HCl-mediated decomposition as mentioned above.

Given the wide variety of known rhodamines, we evaluated the tolerance of substrates with oxygen (**1**–**6**, **9**, **10**) and dimethylsilyl (**7** and **8**) at the 10′-position to represent the most common rhodamines utilized for microscopy. Diazoketones could be reliably formed in each case, but dimethylsilyl substrates were obtained with slightly eroded yields. To ensure that the KF was not interacting with the silicon, the reaction was attempted without KF and there was no improvement in yield.

To assess the optimal route for photoactivatable dyes with covalent protein tags, we installed the diazoketone both before and after coupling of a HaloTag to the dye. In the case of both silicon (**7**) and oxygen (**9**) xanthenes, we observed that yields were higher before conjugation of the tag compared to direct diazoketone installation on the HaloTagged dye (**6b**, **8b**, and **10c**). It is possible that the cause of the eroded yield is the PEG-like moiety on the HaloTag which could render the compound hygroscopic. Fortunately, the higher yielding route is more modular as it allows late-stage conjugation of other tags such as CLIP-tag (**10b**) to accommodate any engineered protein system.

**Scheme 4:**
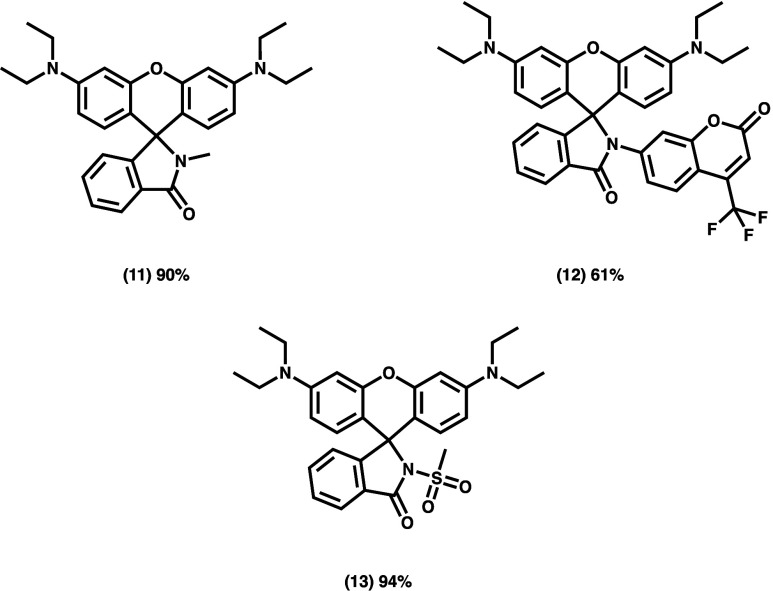
Substrate Scope of Nucleophiles Reacted with **1e** To Give Rhodamine Derivatives

Finally, to explore the value of this method for other applications, we subjected the Ghosez-derived acid chloride to nucleophiles beyond TMSCHN_2_ (Scheme 4). Methyl amine rapidly reacted and formed the colorless *leuco* form almost immediately giving **11** in high yields. Less nucleophilic amines also reacted readily to give aniline-derived lactam substituents. For example, 4-amino-7-trifluoromethylcoumarin rapidly forms the corresponding lactam, **12**, of a photochromic chemotype previously described by Lavis and co-workers.^25^ Weaker nucleophiles, such as methyl sulfonamide, **13**, were also tolerated by this method. Taken together, these indicate that the method provides a strategy to rapidly and easily tune the open-closed equilibrium of rhodamines to suit the needs of the end user by altering the nucleophilicity of the ring-closing atom.

### Application of Novel Diazoketone to Observe Distinct Focal Adhesion Regions in β-Catenin Dynamics

We recently introduced a high-throughput single-molecule tracking system which permits us to evaluate protein dynamics in living cells on an unprecedented scale and shows how this platform could be used in service of systems biology and drug discovery initiatives.^18^ Nonspecific labeling determined by measurement of fluorophore detections of JF549-HaloTag in HaloTag protein-negative cells generated substantial noise from our protein dynamics measurements. To combat this, we employed our improved synthetic route to generate novel photoactivatable dyes with enhanced physicochemical properties.

We found that installation of a dimethylcarboxamide at the 2-position of the azetidine (**10c**) increased solubility and passive permeability across cell membranes, resulting in a 14-fold reduction of nonspecific labeling in U2OS cells containing no HaloTag protein compared to photoactivatable JF549-HaloTag (Figure 1A, Table S3). To assess the value of our improved dye, we labeled U2OS cells bearing an endogenous knock-in of HaloTag fusion to β-catenin, a protein with multiple cellular roles, including the activation of Wnt-responsive gene pathways via transcription activation. β-Catenin is a protein of intense interest because of its role in diseases such as colorectal cancer. Cells were labeled with 50 nM **10c**, then imaged under conditions where the positions of individual molecules could be identified with subpixel precision, and tracked in the live cell. As reported, the α-diazoketone was sensitive to 405 nm laser uncaging.^14,17^ Upon irradiation of cells incubated with **10c** at 1 mW (Figure 1B, frames 250–750), we observed an increase in quantity of detectable fluorophores—an indication of photoactivation, which leveled off suggesting an equilibration of the rate of photobleaching and activation. Upon increasing the irradiation power to 2 mW (Figure 1B, frames 750–1500), the activation rate increased, and the quantity of detectable fluorophores followed suit. In contrast to the 1 mW irradiation, the rate of photobleaching outpaced the activation around frame 1000 resulting in a time dependent reduction in quantity of fluorophores detected from frames 1000–1500. We then repeated this process twice at 8 mW (Figure 1B, frame 1500–2500) and 16 mW (Figure 1B, frames 2500–5000) to show that this process could be repeated and imaging could be conducted until essentially all of the dye was uncaged. After localization and tracking, we built a profile of the dynamic states present in our sample. We found that there were at least three distinct dynamical states, ranging from very slow diffusing (<0.1 μm^2^/sec) to very fast diffusing (>5 μm^2^/sec), consistent with the many reported functional roles of β-catenin in the cell (Figure 1C).^26^ Because of the photoactivatable nature of **10c**, we could observe thousands of protein trajectories per cell and generate individual super-resolved images of β-catenin for each of the dynamical states we observed (Figure 1D, left). Interestingly, we discovered that the different dynamical states correspond also to spatially distinct regions of the cell. The slowest population which moves at <0.1 μm^2^/s (represented in magenta in Figure 1C and Figure 1D) is found almost entirely in the plasma membrane of the cell. The middle population which diffuses at 0.1–1 μm^2^/s (represented as green in Figure 1C and Figure 1D) is also associated with the plasma membrane of the cell but can be inferred to be engaging in cell–cell gap junctions owing to its faster rate of diffusion. By contrast, the fastest populations which diffuse at >1 μm^2^/s (represented as blue in Figure 1C and Figure 1D) are predominantly cytoplasmic and nuclear, consistent with the role of β-catenin in activity of Wnt-responsive genes in the nucleus.

## Conclusion

Previously, the synthesis of cyclic diazoketones in the presence of azetidines has been challenging due to acid-assisted nucleophilic azetidine opening. Here, we have described a method compatible with azetidines by using Ghosez’s reagent which does not produce acid as a byproduct. This method has facilitated synthesis of challenging targets such as PA-rhodamines with [1.1.3] bridged bicyclic azetidines (**6b**), which were previously inaccessible. Beyond azetidine-containing dyes, we have demonstrated that this method can be used to routinely access rhodamines with amides and sulfonamides on the southern arene, which has been a fairly underexplored area. We were also able to access **10c** which is amenable to our high-throughput SMT screening platform. With it, we were also able to observe distinct focal adhesion regions in β-catenin dynamics, indicating that the method can yield novel dyes and can be applied in interesting biological settings. Further characterization and detailed accounts of our novel applications of our dyes presented here and more dyes accessed via this method will be disclosed in a later publication.

### General Procedure for Synthesis of Photoactivatable Rhodamines

To an oven-dried 20 mL vial with a septum cap was added 3′,6′-bis(diethylamino)-3*H*-spiro[2-benzofuran-1,9′-xanthen]-3-one (**1a**) (1.00 g, 2.26 mmol, 1 equiv) and anhydrous CH_2_Cl_2_ (20 mL). To the resulting solution was added anhydrous MeCN (20 mL) followed by 4 Å molecular sieves (2.41 g) and CaO (380 mg, 6.78 mmol, 3 equiv). The reaction mixture was stirred for 10 min before Ghosez’s reagent (1.2 mL, 9.04 mmol, 4 equiv) was added dropwise, causing the solution to darken in color. The reaction was monitored by treating an aliquot of the reaction mixture with methylamine (2 M in THF) followed by LC-MS analysis. Once the starting material was entirely consumed and the corresponding acid chloride (represented by the methyl amide) had formed (usually 2 min or less), TMSCHN_2_ (9.04 mL 2 M in diethyl ether, 18.1 mmol, 8 equiv) and KF (1.05 g, 18.1 mmol, 8 equiv) were added sequentially. Reaction was allowed to continue stirring at room temperature until all acid chloride was consumed as determined by LC-MS (usually less than 1 h). Note, while LC-MS was used in our laboratory, treatment with methylamine is also easily monitored by TLC as a dark spot under UV irradiation. Synthesis of Compound-11 (vide infra) or the analogous compound for the end user’s dye can be used to generate a standard for facile TLC reaction monitoring. The reaction mixture was then concentrated directly onto Celite and purified by normal phase flash chromatography on 24 g RediSep basic alumina columns (∼10 g silica per mmol starting material) using a gradient of 0–20% EtOAc in Toluene. ***Caution!*** TMSCHN_2_ is an inhalation hazard and should be handled with elevated levels of care as inhalation of TMCHN_2_ can cause fatal respiratory toxicity including pulmonary edema. A well-ventilated fume hood should be used at all times.^27^

## Data Availability

The data underlying this study are available in the published article and it Supporting Information.

## Supporting Information Available

Experimental details, procedures, and chemical and biological characterization data (PDF)
