# Supplementary material for: Optimized Properties and Synthesis of Photoactivatable Diazoketorhodamines Facilitate and Enhance High-Throughput Single-Molecule Tracking
Source: J Org Chem. 2024 Jun 5;89(20):14658–64. doi: 10.1021/acs.joc.4c00718 (PMC11494646; doi:10.1021/acs.joc.4c00718)
Supplement: Supplementary file 1 — jo4c00718_si_001.pdf [file jo4c00718_si_001.pdf]

Electronic Supplementary Information

Optimized Properties and Synthesis of Photoactivatable Diazoketorhodamines Facilitate and Enhance High Throughput Single Molecule Tracking

Nicholas W. Pino, [a]* Anne R. Sizemore,[a] Leah Cleary,[a] Helen Liu,[a] David T. McSwiggen,[a] Dan Song,[a] Hilary P. Beck,[a] Kylie Cheng,[a] Miki Hardy,[a] Jessica Hsiung,[a] Yangzhong Tang,[a] Rajender Anugula,[b] Santhosh Lakshman,[b] Ravi K. Merneedi,[b] Pradipta Sinha,[b]

[a] Eikon Therapeutics Inc., Hayward, CA
[b] Aragen Life Sciences Ltd., Bengaluru, India
* Corresponding author: pinon@eikontx.com

LIST OF CONTENTS

1. MATERIALS AND METHODS **S2**

2. GENERAL SYNTHESES OF PHOTOACTIVATABLE RHODAMINES AND ADDITION OF NUCLEOPHILES INTO ACYL CHLORIDES **S2**

3. SYNTHESIS **S2**

4. ANALYTICAL DATA **S18**

5. SINGLE MOLECULE TRACKING SAMPLE PREPARATION **S25**

6. SINGLE MOLECULE TRACKING IMAGE ACQUISITION **S25**

7. SINGLE MOLECULE TRACKING IMAGE ANALYSIS **S25**

8. EXTENDED OPTIMIZATION DATA **S26**

9. SOLUBILITY AND PERMEABILITY DATA **S27**

10. REFERENCES **S27**

1. MATERIALS AND METHODS

Commercially obtained reagents were used as received except where indicated otherwise. 4 Å molecular sieves were purchased from Fisher Scientific and were pulverized with a pestle and mortar, dried in a vacuum oven, and stored in a desiccator for all uses. Calcium oxide, Celite®, and Sodium sulfate were purchased from Fisher Scientific. Ghosez' reagent, $Pd_2(dba)_3$, Xphos, Deuterated NMR solvents, and DIPEA were purchased from Sigma Aldrich. Methylamine, Potassium fluoride, Cesium carbonate, Lithium hydroxide monohydrate, T3P, TBTU, and HOBt were purchased from AK Scientific. $TMSCHN_2$ was purchased from Oakwood Chemicals. 4-trifluoromethyl-7-aminocoumarin, Methylsulfonamide, and 4-((4-(aminomethyl)benzyl)oxy)pyrimidin-2-amine were purchased from Ambeed. Sulfuric acid was purchased from VWR. Azetidine was purchased from Alfa Aesar. 1-[2-(2-aminoethoxy)ethoxy]-6-chlorohexane was purchased from WuxiAppTec. N,N-Dimethylazetidine-3-carboxamide hydrochloride was purchased from Combi-Blocks. Solvents were purchased from Thermo Scientific used as received. Reactions were stirred and heated (when applicable) using IKA RCT basic heating and stirring plates and IKA heating blocks monitored by thermocouple. Reactions were monitored by LC-MS or thin layer chromatography under short and long wave UV-irradiation. LC-MS data was collected on an Agilent 1260 LC-MS. Normal phase column chromatography was conducted on Teledyne ISCO Combiflash NextGen 300 or 300+ chromatography machines. 24 g RediSep® Basic alumina columns were purchased from Teledyne ISCO and were rated to have a particle size of 20-64 μm, mesh size of 230-400, proe size of 60 Å, surface area of 200 ± 50 m^2/g, pH of 9.7 ± 0.3, and loading capacity of 0.5 – 4%. Reverse phase HPLC was conducted on Teledyne ISCO AccqPrep HP 150 HPLC machines with a Kinetex® 5 μm XB-C18 100 Å, dimensions: 50 x 30 mm. ^1H and $^{13}C\{^1H\}$ NMR data were collected on Bruker Ascend 400 or collected by Aragen Life Sciences (Bengaluru, India). Data for ^1H and $^{13}C\{^1H\}$ NMR are reported relative to residual solvent as calculated by MestreNova and are reported in the following format: ppm (multiplicity, coupling constant, integration). HRMS data were collected with a Thermo Q-Exactive Hybrid Quadrupole-Orbitrap in ESI positive mode by Lotus Separations (Princeton, NJ). Solubility and permeability data were collected by Pharmaron, Inc (Beijing, China).

GENERAL SYNTHESES OF PHOTOACTIVATABLE RHODAMINES AND ADDITION OF NUCLEOPHILES INTO ACYL CHLORIDES

General procedure for addition of nucleophiles into acyl chloride

To an oven-dried 20 mL vial with a septum cap was added 3',6'- bis(diethylamino)-3H-spiro[2-benzofuran-1,9'-xanthen]-3-one (93 mg, 210 μmol, 1 equiv) and taken up in CH_2Cl_2 (20 mL). To the resulting solution was added MeCN (20 mL) followed by 4 Å molecular sieves (213 mg) and CaO (35 mg, 629 μmol, 3 equiv). The reaction mixture was stirred for 10 minutes before Ghosez' reagent (111 μL, 839 μmol, 4 equiv) was added dropwise, causing the solution to darken in color. To the reaction mixture was added 4-trifluoromethyl-7-aminocoumarin (192 mg, 839 μmol, 4 equiv). The reaction was allowed to continue stirring at room temperature until all acid chloride was consumed as determine by LC-MS (usually less than 1 hour). The reaction mixture was then concentrated directly onto Celite® and purified by normal phase flash chromatography on silica gel. **Caution!** $TMSCHN_2$ is an inhalation hazard and should be handled with elevated levels of care as inhalation of $TMCHN_2$ can cause fatal respiratory toxicity including pulmonary edema. A well-ventilated fume hood should be used at all times.[1]

2. SYNTHESIS

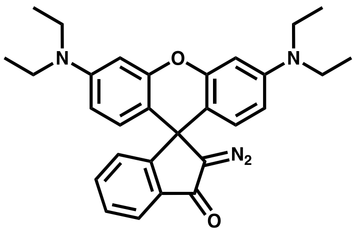

2-diazo-3',6'-bis(diethylamino)spiro[indene-1,9'-xanthen]-3(2*H*)-one (1b). Synthesized according to general procedure for synthesis of photoactivatable rhodamines. Product obtained as an off-white powder. 413 mg, 40% yield. ^{1}H NMR (400 MHz, $CDCl_3$) δ 7.66 (d, *J* = 7.6 Hz, 1H), 7.31 (t, *J* = 7.4 Hz, 1H), 7.23 (t, *J* = 7.5 Hz, 1H), 6.94 (d, *J* = 7.7 Hz, 1H), 6.53 (d, *J* = 8.7 Hz, 2H), 6.25 – 6.20 (m, 2H), 6.14 (d, *J* = 8.9 Hz, 2H), 3.18 (q, *J* = 7.2 Hz, 8H), 1.01 (t, *J* = 7.1 Hz, 13H). ^{13}C{^{1}H} NMR (101 MHz, $CDCl_3$) δ 187.53, 156.50, 152.51, 148.40, 134.61, 134.52, 128.79, 128.15, 125.51, 122.09, 108.12, 107.68, 98.19, 77.35, 77.23, 77.03, 76.72, 49.13, 44.37, 12.66. LCMS m/z (ESI) calcd for [$C_{29}H_{30}N_4O_2$] [M+H]$^+$: 467.59, found: 467.30. Characterization data obtained are in agreement with those previously reported.[2] Purified by normal phase flash chromatography on 24 g RediSep® basic alumina columns (~10 g silica per mmol starting material) using a gradient of 0-20% EtOAc in Toluene.

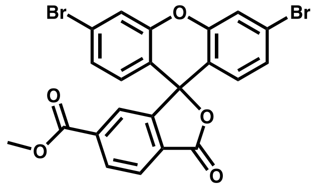

methyl 3',6'-dibromo-3-oxo-3*H*-spiro[isobenzofuran-1,9'-xanthene]-6-carboxylate (S1). Synthesized as reported previously.[3,4] To a stirred solution of 3',6'-dibromo-3-oxo-3H-spiro[isobenzofuran-1,9'-xanthene]-6-carboxylic acid (5 g, 9.95 mmol) in methanol (100 mL) was added conc. sulfuric acid (5 mL) drop wise at 0°C and the reaction mixture was allowed to stir at 80°C for 48 hours. After completion of reaction as determined by LC-MS, reaction mixture was filtered and washed with methanol (20 mL). Filtrate was then concentrated to give crude product. The obtained crude compound was purified by trituration with diethyl ether and *n*-pentane to afford methyl 3',6'-dibromo-3-oxo-3H-spiro[isobenzofuran-1,9'-xanthene]-6-carboxylate (3.5 g, 69%) as an off white solid. ^{1}H NMR (400 MHz, DMSO-d_6): δ 8.27 (d, *J* = 7.6 Hz, 1H), 8.19 (d, *J* = 8 Hz, 1H), 7.90 (s, 1H), 7.71 (s, 2H), 7.33 (d, *J* = 8.8 Hz, 2H), 6.87 (d, *J* = 8.8 Hz, 2H), 3.81 (s, 3H). LCMS m/z (ESI) calcd for [$C_{22}H_{12}Br_2O_5$] [M+H]$^+$: 514.91, found: 514.90. Characterization data obtained are in agreement with those previously reported.[5]

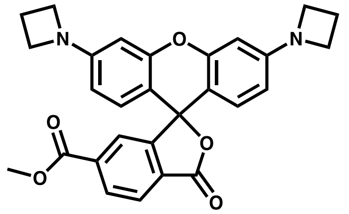

methyl 3',6'-di(azetidin-1-yl)-3-oxo-3*H*-spiro[isobenzofuran-1,9'-xanthene]-6-carboxylate (2a). Synthesized as reported previously.[3,4] To a stirred solution of methyl 3',6'-dibromo-3-oxo-3H-spiro[isobenzofuran-1,9'-xanthene]-6-carboxylate (**S1**) (2.5 g, 4.84 mmol) and azetidine (0.85 g, 14.53 mmol) in anhydrous 1,4-dioxane (50 mL) was added cesium carbonate (5 g, 14.53 mmol). The resulting mixture was sparged with argon for 10 minutes followed by the addition of $Pd_2(dba)_3$ (450 mg, 0.48 mmol) and Xphos (700 mg, 1.45 mmol). The reaction mixture was again sparged with argon gas for 10 min. The reaction mixture was then warmed to 90°C and stirred for 16 hours. After completion of reaction as determined by LC-MS reaction mixture was filtered through a pad of Celite®, washed with methanol (200 mL) and concentrated. The obtained crude compound was purified by reverse phase column chromatography using

a gradient of 20-100% acetonitrile in water with 10 mM ammonium acetate to afford of methyl 3',6'-di(azetidin-1-yl)-3-oxo-3H-spiro[isobenzofuran-1,9'-xanthene]-6-carboxylate (1.1 g, 50%)) as a violet solid. ^{1}H NMR (400 MHz, DMSO-d_6): δ 8.31 (s, 2H), 7.92 (s, 1H), 7.12-6.91 (m, 2H), 6.68-6.52 (m, 4H), 4.39-4.19 (m, 8 H), 3.88 (s, 3H), 2.49-2.39 (m, 4H). LCMS m/z (ESI) calcd for [$C_{28}H_{24}N_2O_5$] [M+H]$^+$: 469.17, found: 469.60. Characterization data obtained are in agreement with those previously reported.[5]

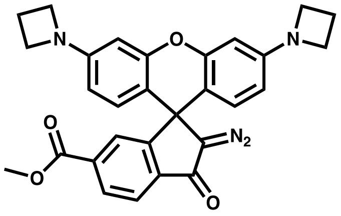

methyl 3',6'-di(azetidin-1-yl)-2-diazo-3-oxo-2,3-dihydrospiro[indene-1,9'-xanthene]-6-carboxylate (2b). Synthesized according to general procedure for synthesis of photoactivatable rhodamines. Product obtained as an off-white powder. 23 mg, 44% yield. ^{1}H NMR (400 MHz, Pyr-d5) δ 8.14 (d, J = 8.0 Hz, 1H), 8.08 (d, J = 8.0 Hz, 1H), 8.02 (s, 1H), 6.98 (d, J = 8.5 Hz, 2H), 6.35 (d, J = 2.3 Hz, 2H), 6.19 (dd, J = 8.5, 2.4 Hz, 2H), 3.68 (t, J = 7.8 Hz, 8H), 3.59 (d, J = 2.5 Hz, 3H), 2.04 (p, J = 7.3 Hz, 4H).^{13}C{^{1}H} NMR (101 MHz, Pyr-d5) δ 185.51, 165.51, 156.72, 153.08, 152.39, 150.00, 149.94, 149.66, 138.27, 136.05, 135.51, 135.26, 135.02, 129.90, 128.50, 126.45, 123.51, 123.26, 123.02, 122.50, 109.04, 108.32, 98.53, 76.77, 66.96, 51.94, 49.75, 16.58.

LCMS m/z (ESI) calcd for [$C_{29}H_{24}N_4O_4$] [M+H]$^+$: 493.54, found: 493.20 Characterization data obtained are in agreement with those previously reported.[5] Purified by normal phase flash chromatography on 8 g RediSep® basic alumina columns using a gradient of 0-20% EtOAc in Toluene.

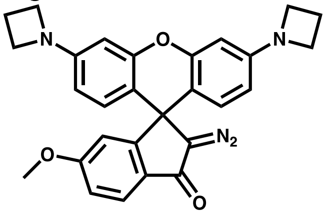

3',6'-di(azetidin-1-yl)-2-diazo-6-methoxyspiro[indene-1,9'-xanthen]-3(2*H*)-one (3b). Synthesized according to general procedure for synthesis of photoactivatable rhodamines. Product obtained as an off-white powder. 25 mg, 50% yield. ^{1}H NMR (400 MHz, $CDCl_3$) δ 7.27 (d, J = 2.5 Hz, 1H), 7.04 (dd, J = 8.5, 2.6 Hz, 1H), 6.94 (d, J = 8.5 Hz, 1H), 6.68 (d, J = 8.5 Hz, 2H), 6.15 (d, J = 2.4 Hz, 2H), 6.08 (dd, J = 8.5, 2.4 Hz, 2H), 3.91 – 3.83 (m, 12H), 2.36 (p, J = 7.2 Hz, 5H). ^{13}C{^{1}H} NMR (101 MHz, Pyr-d5) δ 185.7, 165.7, 159.3, 152.9, 152.3, 128.7, 127.5, 123.9, 115.9, 109.9, 109.8, 108.2, 98.4, 75.3, 55.3, 51.9, 49.2, 29.7, 16.6. LCMS m/z (ESI) calcd for [$C_{28}H_{24}N_4O_3$] [M+H]$^+$: 465.53, found: 465.20. Characterization data obtained are in agreement with those previously reported.[5] Purified by normal phase flash chromatography on 8 g RediSep® basic alumina columns using a gradient of 0-20% EtOAc in Toluene.

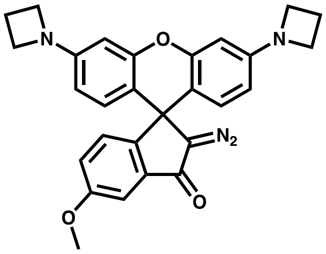

3',6'-di(azetidin-1-yl)-2-diazo-5-methoxyspiro[indene-1,9'-xanthen]-3(2*H*)-one (4b). Synthesized according to general procedure for synthesis of photoactivatable rhodamines. Product obtained as a clear greenish film. 25 mg, 50% yield. ^{1}H NMR (400 MHz, $CDCl_3$) δ 7.59 (d, J = 8.5 Hz, 1H), 6.77 (dd, J = 8.5, 2.3

Hz, 1H), 6.57 (d, *J* = 8.5 Hz, 2H), 6.32 (d, *J* = 2.3 Hz, 1H), 6.01 (d, *J* = 2.3 Hz, 2H), 5.94 (dd, *J* = 8.5, 2.4 Hz, 2H), 3.73 (t, *J* = 7.2 Hz, 8H), 3.54 (s, 3H), 2.21 (p, *J* = 7.3 Hz, 4H). $^{13}C\{^1H\}$ NMR (101 MHz, $CDCl_3$) δ 186.4, 165.4, 158.8, 152.6, 151.9, 128.7, 127.4, 123.8, 115.8, 109.8, 109.3, 107.9, 98.2, 77.4, 55.6, 52.2, 48.9, 29.7, 16.8. LCMS m/z (ESI) calcd for $[C_{28}H_{24}N_4O_3]$ $[M+H]^+$: 465.53, found: 465.20. Characterization data obtained are in agreement with those previously reported.[5] Purified by normal phase flash chromatography on 8 g RediSep® basic alumina columns using a gradient of 0-20% EtOAc in Toluene.

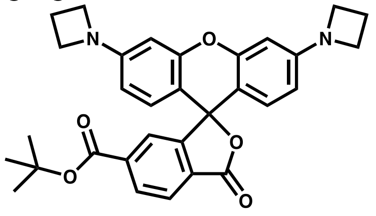

***tert*-butyl 3',6'-di(azetidin-1-yl)-3-oxo-3*H*-spiro[isobenzofuran-1,9'-xanthene]-6-carboxylate (5a).** Synthesized as reported previously.[4] To a stirred solution of tert-butyl 3',6'-dibromo-3-oxo-3H-spiro[2-benzofuran-1,9'-xanthene]-6-carboxylate (50 mg, 0.090 mmol), $Pd_2(dba)_3$ (8.2 mg, 0.009 mmol), XPhos (12.8 mg, 0.09 mmol), and Cs_2CO_3 (81.7 mg, 0.251 mmol) in anhydrous 1,4-dioxane (1 mL) was added azetidine (0.013 mL, 0.197 mmol). The resulting suspension was heated to 100 °C for 3 hours. The reaction mixture was then diluted with methanol and concentrated directly onto silica for normal phase column chromatography using a gradient of 1-5% MeOH in CH_2Cl_2 which gave the product as a dark purple solid. LCMS m/z (ESI) calcd for $[C_{31}H_{30}N_2O_5]$ $[M+H]^+$: 511.22, found: 511.20. Characterization data obtained are in agreement with those previously reported.[4]

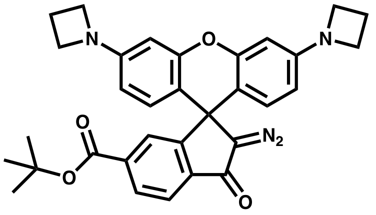

***tert*-butyl 3',6'-di(azetidin-1-yl)-2-diazo-3-oxo-2,3-dihydrospiro[indene-1,9'-xanthene]-6-carboxylate (5b).** Synthesized according to general procedure for synthesis of photoactivatable rhodamines. Product obtained as a white solid. 16 mg, 31% yield. ^1H NMR (400 MHz, Pyr-d5) δ 7.95 – 7.83 (m, 3H), 6.73 (d, *J* = 8.5 Hz, 2H), 6.08 (d, *J* = 2.3 Hz, 2H), 5.89 (dd, *J* = 8.5, 2.4 Hz, 2H), 4.67 (s, 3H), 3.41 (dq, *J* = 11.0, 7.2 Hz, 9H), 1.78 (p, *J* = 7.2 Hz, 4H), 1.10 (s, 9H). $^{13}C\{^1H\}$ NMR (101 MHz, Pyr-d5) δ 185.6, 164.3, 156.8, 153.0, 152.4, 149.4, 138.1, 138.0, 135.5, 129.9, 128.5, 126.3, 109.1, 108.3, 98.5, 81.6, 76.8, 51.9, 49.8, 27.5, 16.5. LCMS m/z (ESI) calcd for $[C_{32}H_{30}N_4O_4]$ $[M+H]^+$: 535.23, found: 535.30. Purified by normal phase flash chromatography on 8 g RediSep® basic alumina columns using a gradient of 0-20% EtOAc in Toluene.

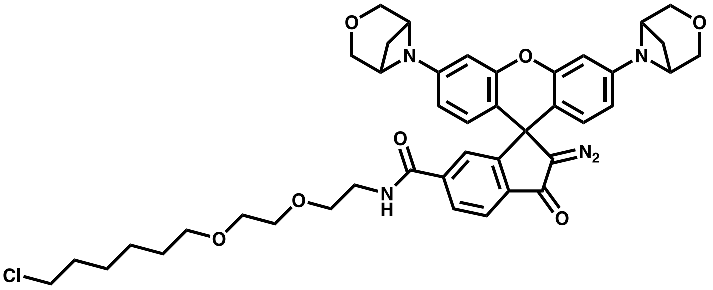

3',6'-di(3-oxa-6-azabicyclo[3.1.1]heptan-6-yl)-*N*-(2-(2-((6-chlorohexyl)oxy)ethoxy)ethyl)-2-diazo-3-oxo-2,3-dihydrospiro[indene-1,9'-xanthene]-6-carboxamide (6b). Synthesized according to general procedure for synthesis of photoactivatable rhodamines. Product obtained as a white solid. 3.5 mg, 12% yield. ^{1}H NMR (400 MHz, Pyr-d5) δ 8.11 (dd, *J* = 7.9, 1.4 Hz, 1H), 8.03 (s, 1H), 7.84 (d, *J* = 7.9 Hz, 1H), 6.73 (d, *J* = 8.4 Hz, 2H), 6.24 (d, *J* = 2.2 Hz, 2H), 6.07 (dd, *J* = 8.5, 2.2 Hz, 2H), 4.08 (dd, *J* = 22.2, 10.6 Hz, 4H), 3.90 (d, *J* = 6.0 Hz, 4H), 3.48 – 3.30 (m, 8H), 3.29 – 3.18 (m, 7H), 3.06 (t, *J* = 6.5 Hz, 2H), 2.34 (d, *J* = 6.7 Hz, 2H), 1.53 (d, *J* = 7.9 Hz, 2H), 1.40 – 1.29 (m, 2H), 1.20 (t, *J* = 7.0 Hz, 2H), 0.99 (q, *J* = 8.0 Hz, 5H). ^{13}C{^{1}H} NMR (101 MHz, Pyr-d5) δ 185.7, 166.1, 156.2, 152.6, 148.7, 141.5, 137.2, 128.7, 128.6, 124.7, 122.7, 122.3, 111.4, 110.6, 102.1, 76.8, 70.8, 70.3, 70.1, 69.7, 62.7, 62.6, 61.5, 49.6, 45.3, 40.3, 32.5, 29.6, 27.9, 26.6, 25.4. HRMS m/z (ESI) calcd for [$C_{46}H_{58}ClN_7O_6Si$] [M+H]$^+$: 768.3164, found: 768.3156. Purified by normal phase flash chromatography on 8 g RediSep® basic alumina columns using a gradient of 0-20% EtOAc in Toluene.

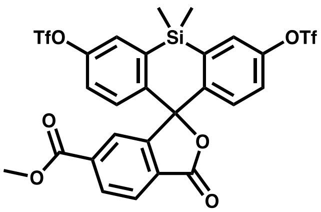

methyl 5,5-dimethyl-3'-oxo-3,7-bis(((trifluoromethyl)sulfonyl)oxy)-3'*H*,5*H*-spiro[dibenzo[*b*,*e*]siline-10,1'-isobenzofuran]-6'-carboxylate (S2). Synthesized as reported previously.[3,4] To a stirred solution of 5,5-dimethyl-3'-oxo-3,7-bis(((trifluoromethyl)sulfonyl)oxy)-3'H,5H-spiro[dibenzo[b,e]siline-10,1'-isobenzofuran]-6'-carboxylic acid (0.9 g, 1.319 mmol) in THF (18 mL) and methanol (4.5 mL) was added trimethylsilyl diazomethane 2M in Et_2O (2.0 mL, 3.956 mmol) drop wise at 0 °C under argon atmosphere. The reaction mixture was then allowed to warm to room temperature and stirred for 15 min. Upon completion of the reaction as monitored determined by TLC, the reaction was concentrated and purified by silica gel column chromatography with a gradient of 0-15% ethyl acetate in petroleum ether to afford methyl 5,5-dimethyl-3'-oxo-3,7-bis(((trifluoromethyl)sulfonyl)oxy)-3'H,5H-spiro[dibenzo[b,e]siline-10,1'-isobenzofuran]-6'-carboxylate (0.75 g , 81% yield) as a white solid. ^{1}H NMR (400 MHz, $CDCl_3$) δ 8.22 (dd, *J* = 8.0, 1.2 Hz, 1H), 8.05 – 7.95 (m, 2H), 7.51 (d, *J* = 2.6 Hz, 2H), 7.21 – 7.10 (m, 4H), 3.88 (s, 3H), 0.74 (s, 3H), 0.64 (s, 3H). LCMS m/z (ESI) calcd for [$C_{26}H_{18}F_6O_{10}S_2Si$] [M+H]$^+$: 697.00, found: 697.44. Characterization data obtained are in agreement with those previously reported.[4]

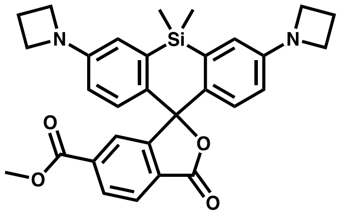

methyl 3,7-di(azetidin-1-yl)-5,5-dimethyl-3'-oxo-3'*H*,5*H*-spiro[dibenzo[*b*,*e*]siline-10,1'-isobenzofuran]-6'-carboxylate (7a). Synthesized as reported previously.[3,4] To a stirred solution of methyl 5,5-dimethyl-3'-oxo-3,7-bis(((trifluoromethyl)sulfonyl)oxy)-3'H,5H-spiro[dibenzo[b,e]siline-10,1'-isobenzofuran]-6'-carboxylate (0.75 g, 1.077 mmol) in anhydrous 1,4-dioxane (19 mL) was added tris(dibenzylideneacetone)dipalladium(0) (99 mg, 0.108 mmol), Xphos (154 mg, 0.323 mmol) and cesium carbonate (1.052 g, 3.23 mmol) and degassed with argon for 15 min. Azetidine (184 mg, 3.23 mmol) was then added before the reaction mixture was sealed and warmed to 100 °C and stirred for 3 hours. After completion of the reaction as determined by TLC, the reaction mixture was cooled to room temperature and filtered through a Celite® pad before being washed with ethyl acetate and concentrated and purified by silica gel column chromatography to afford methyl 3,7-di(azetidin-1-yl)-5,5-dimethyl-3'-oxo-3'H,5H-

spiro[dibenzo[b,e]siline-10,1'-isobenzofuran]-6'-carboxylate (0.4 g , 73% yield) as pale green solid. ^{1}H NMR (400 MHz, DMSO-d_6): δ 8.19 (dd, J = 8.0 Hz, 0.8 Hz, 1H), 8.0 (d, J = 8.0, 1H), 7.35 (s, 1H), 6.76 (d, J = 8.8 Hz, 2H), 6.66 (d, J =2.4 Hz, 2H), 6.27 (dd, J =8.4 Hz, 2.4 Hz, 2H), 3.91 (t, J = 7.2 Hz, 11H), 2.39 – 2.32 (m, 4H), 0.64 (d, J =26 Hz, 6H). LCMS m/z (ESI) calcd for [$C_{30}H_{30}N_2O_4Si$] [M+H]$^+$: 511.20, found: 511.86. Characterization data obtained are in agreement with those previously reported.[4]

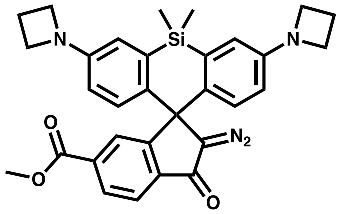

methyl 3,7-di(azetidin-1-yl)-2'-diazo-5,5-dimethyl-3'-oxo-2',3'-dihydro-5*H*-spiro[dibenzo[*b*,*e*]siline-10,1'-indene]-6'-carboxylate (7b). Synthesized according to general procedure for synthesis of photoactivatable rhodamines. Product obtained as a yellow solid. 15% yield. LCMS m/z (ESI) calcd for [$C_{31}H_{30}N_4O_3Si$] [M+H]$^+$: 535.21, found: 535.32. Characterization data obtained are in agreement with those previously reported.[2] Purified by normal phase flash chromatography on 8 g RediSep® basic alumina columns using a gradient of 0-20% EtOAc in Toluene.

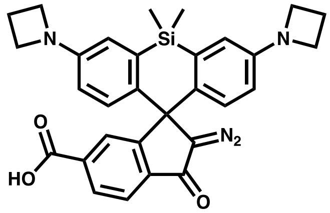

3,7-di(azetidin-1-yl)-2'-diazo-5,5-dimethyl-3'-oxo-2',3'-dihydro-5*H*-spiro[dibenzo[*b*,*e*]siline-10,1'-indene]-6'-carboxylic acid (S3). To a stirred solution of methyl 3,7-di(azetidin-1-yl)-2'-diazo-5,5-dimethyl-3'-oxo-2',3'-dihydro-5H-spiro[dibenzo[b,e]siline-10,1'-indene]-6'-carboxylate (40 mg, 0.075 mmol) in THF (2 mL), methanol (2 mL) and water (1 mL) was added lithium hydroxide monohydrate (31 mg, 0.75 mmol) and stirred for 3 hours. The reaction mixture was concentrated to afford 3,7-di(azetidin-1-yl)-2'-diazo-5,5-dimethyl-3'-oxo-2',3'-dihydro-5H-spiro[dibenzo[b,e]siline-10,1'-indene]-6'-carboxylic acid (40 mg, crude) as pale brown solid. This material was used immediately to synthesize **S4** without further purification. LCMS m/z (ESI) calcd for [$C_{30}H_{28}N_4O_3Si$] [M+H]$^+$: 521.19, found: 521.46.

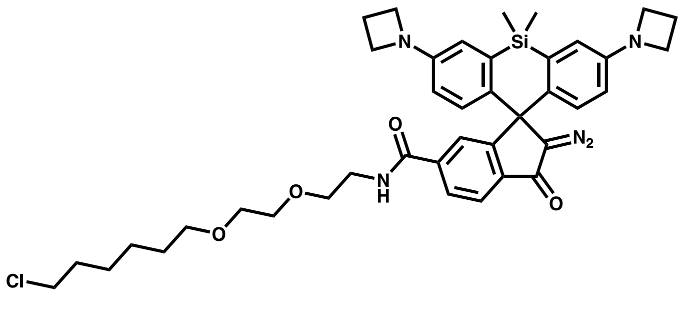

3,7-di(azetidin-1-yl)-*N*-(2-(2-((6-chlorohexyl)oxy)ethoxy)ethyl)-2'-diazo-5,5-dimethyl-3'-oxo-2',3'-dihydro-5*H*-spiro[dibenzo[*b*,*e*]siline-10,1'-indene]-6'-carboxamide (S4). To a stirred solution of 3,7-di(azetidin-1-yl)-2'-diazo-5,5-dimethyl-3'-oxo-2',3'-dihydro-5H-spiro[dibenzo[b,e]siline-10,1'-indene]-6'-carboxylic acid (40 mg, 0.077 mmol) and 2-(2-((6-chlorohexyl)oxy)ethoxy)ethan-1-amine (26 mg, 0.115

mmol) in anhydrous DMF (1.0 mL) was added DIPEA (0.06 mL, 0.307 mmol) and T3P (50% soln. in ethyl acetate, 100 mg, 0.154 mmol) at room temperature under argon atmosphere and stirred for 2 hours. Upon completion of the reaction as determined by TLC, reaction mixture was quenched with water (10 mL). Compound was extracted from aqueous layer with ethyl acetate (20 mL). The organic layer was washed with brine solution (2 x 20 mL) and dried over anhydrous sodium sulfate and concentrated. Organic layer was concentrated and purified by preparative HPLC to afford 3,7-di(azetidin-1-yl)-N-(2-(2-((6-chlorohexyl)oxy)ethoxy)ethyl)-2'-diazo-5,5-dimethyl-3'-oxo-2',3'-dihydro-5H-spiro[dibenzo[b,e]siline-10,1'-indene]-6'-carboxamide (11.8 mg, 22% yield over two steps) as pale yellow solid. ^{1}H NMR (400 MHz, Pyr-d5) δ 9.43 (t, *J* = 5.7 Hz, 1H), 8.32 (d, *J* = 7.9 Hz, 1H), 8.14 (d, *J* = 4.4 Hz, 1H), 7.07 (d, *J* = 8.7 Hz, 2H), 6.77 (d, *J* = 2.6 Hz, 2H), 6.32 (dd, *J* = 8.8, 2.7 Hz, 2H), 3.77 – 3.65 (m, 10H), 3.60 (t, *J* = 5.7 Hz, 2H), 3.49 (q, *J* = 4.6 Hz, 2H), 3.44 (d, *J* = 6.2 Hz, 3H), 3.29 (t, *J* = 6.5 Hz, 2H), 2.08 (p, *J* = 7.2 Hz, 4H), 1.58 (p, *J* = 6.8 Hz, 2H), 1.44 (p, *J* = 6.7 Hz, 2H), 1.24 (q, *J* = 10.3 Hz, 6H), 0.65 (s, 3H), 0.51 (s, 3H). ^{13}C{^{1}H} NMR (101 MHz, Pyr-d5) δ 196.4, 186.9, 166.4, 158.2, 150.4, 141.5, 137.4, 135.8, 133.6, 129.7, 127.8, 124.9, 123.5, 122.7, 115.0, 114.4, 78.6, 70.8, 70.3, 70.1, 69.8, 58.0, 52.0, 45.3, 40.3, 32.5, 26.6, 25.4, 16.8, 0.7, -0.10. LCMS m/z (ESI) calcd for [$C_{40}H_{48}ClN_5O_4Si$] [M+H]$^+$: 726.39, found: 726.54. Characterization data obtained are in agreement with those previously reported.[2] Purified by reverse phase HPLC using a gradient of 20-100% acetonitrile in water.

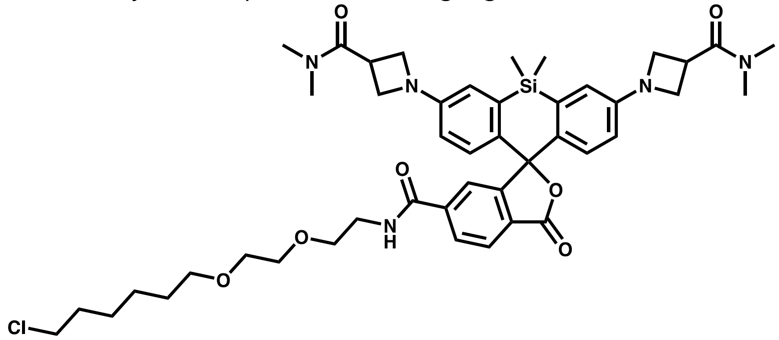

1,1'-(6'-((2-(2-((6-chlorohexyl)oxy)ethoxy)ethyl)carbamoyl)-5,5-dimethyl-3'-oxo-3'*H*,5*H*-spiro[dibenzo[*b*,*e*]siline-10,1'-isobenzofuran]-3,7-diyl)bis(*N,N*-dimethylazetidine-3-carboxamide) (8a). To a solution of 2'-diazo-3,7-bis(3-(dimethylcarbamoyl)azetidin-1-yl)-5,5-dimethyl-3'-oxo-2',3'-dihydro-5*H*-spiro[dibenzo[*b*,*e*]siline-10,1'-indene]-6'-carboxylic acid (20 mg, 0.03 mmol) in DMF (1 mL) was added TBTU (12.1 mg, 0.03mmol), HOBt (7.2 mg, 0.03 mmol) and DIPEA (0.01 mL, 0.06 mmol). The dark blue reaction mixture became light green after 10 minutes when 1-[2-(2-aminoethoxy)ethoxy]-6-chlorohexane (9.8 mg, 0.03 mmol) was added. The mixture was allowed to stir at room temperature for 21 hours until reaction was complete as determined by LC-MS. Reaction was concentrated and purified by reverse phase chromatography using a gradient of 20-100% acetonitrile in water to give a blue-green film. ^{1}H NMR (400 MHz, CD_3OD) δ 8.08 – 7.98 (m, 2H), 7.67 (s, 1H), 6.80 (d, *J* = 2.7 Hz, 2H), 6.74 (d, *J* = 8.7 Hz, 2H), 6.39 (dd, *J* = 8.7, 2.6 Hz, 2H), 4.13 (ddd, *J* = 8.3, 7.0, 1.4 Hz, 4H), 3.98 (td, *J* = 6.7, 3.1 Hz, 4H), 3.89 (d, *J* = 7.3 Hz, 2H), 3.66 – 3.47 (m, 12H), 3.38 (t, *J* = 6.5 Hz, 4H), 3.00 (s, 6H), 2.95 (s, 6H), 1.72 – 1.62 (m, 2H), 1.51 – 1.40 (m, 2H), 1.34 (q, *J* = 7.7 Hz, 2H), 1.28 (m, *J* = 13.1 Hz, 4H), 0.64 (s, 3H), 0.54 (s, 3H). HRMS m/z (ESI) calcd for [$C_{45}H_{57}ClN_4O_8Si$] [M+H]$^+$: 844.3872, found: 844.3863.

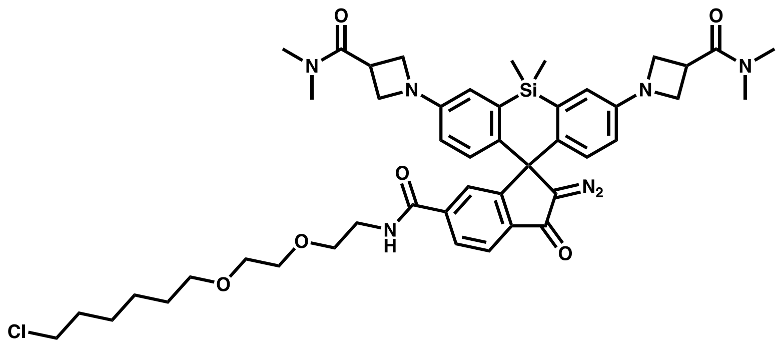

1,1'-(6'-((2-(2-((6-chlorohexyl)oxy)ethoxy)ethyl)carbamoyl)-2'-diazo-5,5-dimethyl-3'-oxo-2',3'-dihydro-5*H*-spiro[dibenzo[*b*,*e*]siline-10,1'-indene]-3,7-diyl)bis(*N,N*-dimethylazetidine-3-carboxamide) (8b). Synthesized according to general procedure for synthesis of photoactivatable rhodamines. Product obtained as a white powder. 1.7 mg, 7% yield. ^{1}H NMR (400 MHz, Pyr-d5) δ 9.28 (s, 1H), 8.10 (d, *J* = 7.7 Hz, 1H), 7.91 (q, *J* = 3.8 Hz, 1H), 6.82 (d, *J* = 8.3 Hz, 2H), 6.59 (t, *J* = 3.8 Hz, 2H), 6.15 (q, *J* = 5.2 Hz, 2H), 3.93 (dq, *J* = 13.9, 6.7 Hz, 4H), 3.81 (td, *J* = 7.7, 3.6 Hz, 4H), 3.48 – 3.31 (m, 7H), 3.21 (dt, *J* = 12.3, 5.9 Hz, 6H), 3.05 (q, *J* = 6.7 Hz, 2H), 2.64 (d, *J* = 6.6 Hz, 6H), 2.43 (d, *J* = 6.9 Hz, 5H), 1.34 (q, *J* = 7.0 Hz, 2H), 1.20 (q, *J* = 6.9 Hz, 2H), 0.99 (s, 3H), 0.39 (d, *J* = 6.9 Hz, 3H), 0.25 (d, *J* = 6.8 Hz, 3H). ^{13}C{^{1}H} NMR (101 MHz, Pyr-d5) δ 171.0, 166.5, 158.1, 155.8, 149.1, 141.6, 134.1, 129.9, 128.0, 122.7, 118.7, 115.1, 114.5, 111.7, 108.3, 78.8, 70.8, 70.3, 70.1, 69.8, 57.9, 54.1, 45.3, 40.3, 36.0, 34.8, 32.7, 29.6, 26.6, 25.4, 0.6, -0.14. HRMS m/z (ESI) calcd for [$C_{46}H_{58}ClN_7O_6Si$] [M+H]$^+$: 868.3985, found: 868.3975. Purified by reverse phase HPLC using a gradient of 20-100% acetonitrile in water.

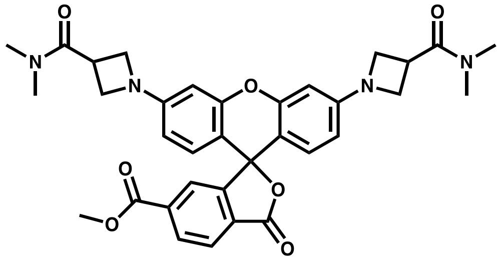

methyl 3',6'-bis(3-(dimethylcarbamoyl)azetidin-1-yl)-3-oxo-3*H*-spiro[isobenzofuran-1,9'-xanthene]-6-carboxylate (9a). To a stirred solution of methyl 3',6'-dibromo-3-oxo-3H-spiro[isobenzofuran-1,9'-xanthene]-6-carboxylate (**S1**) (0.5 g, 0.973 mmol) and *N,N*-dimethylazetidine-3-carboxamide-hydrochloride (0.8 g, 4.86 mmol) in 1,4-dioxane (15 mL) was added cesium carbonate (2.2 g, 6.8 mmol) and reaction mixture was sparged with nitrogen for 20 min. $Pd_2(dba)_3$ (90 mg, 0.09 mmol) and XPhos (140 mg, 0.292 mmol) were added and reaction mixture was again sparged with nitrogen gas for 10 min. The reaction mixture was warmed to 110 °C and stirred for 16 hours. After completion of reaction as determined by LC-MS, reaction mixture was filtered through Celite® pad, washed with methanol (50 mL) and concentrated. The obtained crude compound was purified by reverse phase column chromatography using a gradient of 20-100% acetonitrile in water to afford methyl 3',6'-bis(3-(dimethylcarbamoyl)azetidin-1-yl)-3-oxo-3H-spiro [isobenzofuran-1,9'-xanthene]-6-carboxylate (0.3 g, 51%) as a violet solid. LCMS m/z (ESI) calcd for [$C_{34}H_{34}N_4O_7$] [M+H]$^+$: 611.25, found: 611.42.

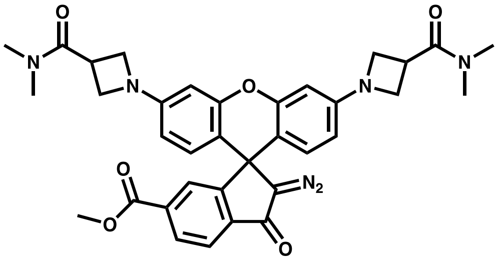

methyl 2-diazo-3',6'-bis(3-(dimethylcarbamoyl)azetidin-1-yl)-3-oxo-2,3-dihydrospiro[indene-1,9'-xanthene]-6-carboxylate (9b). Synthesized according to general procedure for synthesis of photoactivatable rhodamines. Product obtained as a white powder. 5.6 mg, 18% yield. ^{1}H NMR (400 MHz, DMSO-d$_6$): δ 8.03 (d, *J* = 8.0 Hz, 1H), 7.89 (d, *J* = 8.0 Hz, 1H), 7.44 (s, 1H), 6.75 (d, *J* = 8.0 Hz, 2H), 6.23 (s, 2H), 6.16 (d, *J* = 8.4 Hz, 2H), 4.07-4.02 (m, 4H), 3.92-3.79 (m, 6H), 3.77 (s, 3H), 2.88 (s, 6H), 2.84 (s, 6H). LCMS m/z (ESI) calcd for [$C_{35}H_{34}N_6O_6$] [M+H]$^+$: 635.26, found: 635.49. Purified by normal phase flash chromatography on 8 g RediSep® basic alumina columns using a gradient of 0-20% EtOAc in Toluene.

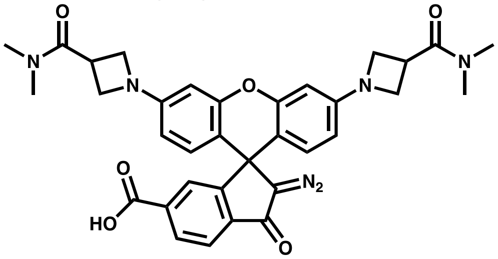

2-diazo-3',6'-bis(3-(dimethylcarbamoyl)azetidin-1-yl)-3-oxo-2,3-dihydrospiro[indene-1,9'-xanthene]-6-carboxylic acid (S5). To a stirred solution of methyl 2-diazo-3',6'-bis(3-(dimethylcarbamoyl)azetidin-1-yl)-3-oxo-2,3-dihydrospiro[indene-1,9'-xanthene]-6-carboxylate (**9**) (0.055 g, 0.086 mmol) in THF (1.0 mL) and MeOH:H_2O (1:1) (1.0 mL) was added lithium hydroxide monohydrate (0.008 g, 0.173 mmol) portion wise at 0 °C. The reaction mixture was stirred for 2 hours at room temperature. After completion of reaction as determined by LC-MS, reaction mixture was concentrate. The obtained crude was purified by trituration with diethyl ether to afford 2-diazo-3',6'-bis(3-(dimethylcarbamoyl) azetidin-1-yl)-3-oxo-2,3-dihydrospiro[indene-1,9'-xanthene]-6-carboxylic acid (0.05 g, 93%) as a brown solid. Product used without further purification. LCMS m/z (ESI) calcd for [$C_{34}H_{32}N_6O_6$] [M+H]$^+$: 621.24, found: 621.60.

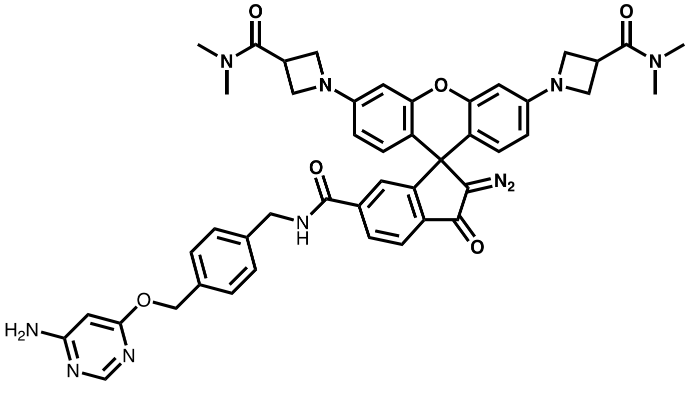

1,1'-(6-((4-(((2-aminopyrimidin-4-yl)oxy)methyl)benzyl)carbamoyl)-2-diazo-3-oxo-2,3-dihydrospiro[indene-1,9'-xanthene]-3',6'-diyl)bis(*N,N*-dimethylazetidine-3-carboxamide) (10b). To a stirred solution of 2-diazo-3',6'-bis(3-(dimethylcarbamoyl)azetidin-1-yl)-3-oxo-2,3-dihydrospiro [indene-1,9'-xanthene]-6-carboxylic acid (**S5**) (0.05 g, 0.081 mmol) and 4-((4-(aminomethyl)benzyl)oxy)pyrimidin-2-amine (0.037 g, 0.161 mmol) in DMF (1 mL) was added *N,N'*-ethyldiisopropylamine (0.06 g, 0.403 mmol) at

0 °C. After 10 min, T3P (50% solution in ethyl acetate; 0.22 g, 0.32 mmol) was added drop wise at 0 °C. The reaction mixture was stirred for 3 hours at room temperature. After completion of reaction as determined by LC-MS, the reaction mixture was concentrated and purified by prep-HPLC using a gradient of 20-100% acetonitrile in water to afford 1,1'-(6-((4-(((2-aminopyrimidin-4-yl)oxy)methyl)benzyl)carbamoyl)-2-diazo-3-oxo-2,3-dihydrospiro[indene-1,9'-xanthene]-3',6'-diyl)bis(*N,N*-dimethylazetidine-3-carboxamide) (0.0156 g, 20%) as pale yellow solid. ^{1}H NMR (400 MHz, DMSO-d$_6$): δ 9.17 (t, *J* = 6.0 Hz, 1H), 8.03-8.01 (dd, *J* = 1.2 Hz, 8.0 Hz, 1H), 7.94 (d, *J* = 5.6 Hz, 1H), 7.85 (d, *J* = 8.0 Hz, 1H), 7.50 (s, 1H), 7.34 (d, *J* = 8.0 Hz, 2H), 7.24 (d, *J* = 8.0 Hz, 2H). 6.70 (d, *J* = 8.8 Hz, 2H), 6.53 (s, 2H), 6.21 (d, *J* = 2.0 Hz, 2H), 6.17-6.14 (dd, *J* = 2.4 Hz, 8.4 Hz, 2H), 8.99 (d, *J* = 5.6 Hz, 1H), 5.23 (s, 2H), 4.38 (d, *J* = 5.6 Hz, 2H), 4.06-4.01 (m, 4H), 3.91 -3.79 (m, 6H), 2.88 (s, 6H), 2.83 (s, 6H). ^{13}C{^{1}H} NMR (101 MHz, Pyr-d5) δ 185.7, 170.9, 169.9, 166.1, 164.7, 159.1, 156.8, 152.3, 152.2, 141.5, 139.2, 137.2, 128.9, 128.7, 128.4, 127.9, 124.5, 109.5, 108.4, 98.7, 97.3, 77.1, 66.8, 54.1, 54.0, 49.7, 43.6, 36.0, 34.9, 32.4. HRMS m/z (ESI) calcd for [$C_{46}H_{44}N_{10}O_6$] [M+H]$^+$: 833.3524, found: 833.3510.

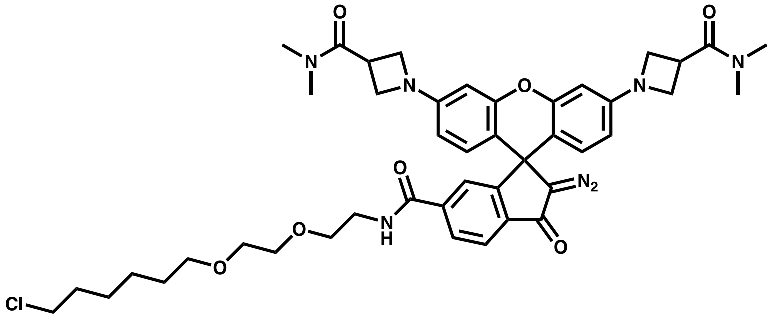

1,1'-(6-((2-(2-((6-chlorohexyl)oxy)ethoxy)ethyl)carbamoyl)-2-diazo-3-oxo-2,3-dihydrospiro[indene-1,9'-xanthene]-3',6'-diyl)bis(*N,N*-dimethylazetidine-3-carboxamide) (10c). Synthesized according to general procedure for synthesis of photoactivatable rhodamines. Product obtained as an off-white film. 1.5 mg, 7% yield. ^{1}H NMR (400 MHz, DMSO-*d$_6$*): δ 8.70 (t, *J* = 5.6 Hz, 1H), 7.99-7.96 (m, 1H), 7.83 (d, *J* = 8.0 Hz, 1H), 7.47 (s, 1H), 6.70 (d, *J* = 8.4 Hz, 2H), 6.22 (d, *J* = 8.4 Hz, 2H), 6.17 (d, J = 8.4 Hz, 2H), 4.04-4.01 (m, 4H), 3.91-3.81 (m. 6H), 3.60 (t, *J* = 6.4 Hz, 2H), 3.46-3.39 (m, 8H), 3.32-3.28 (m, 2H), 2.88 (s, 6H), 2.83 (s, 6H), 1.67-1.63 (m, 2H), 1.42-1.28 (m, 4H), 1.24-1.22 (m, 2H) ppm. ^{13}C{^{1}H} NMR (101 MHz, Pyr-d5) δ 199.4, 185.3, 170.9, 166.3, 161.8, 152.4, 135.3, 135.1, 135.0, 134.8, 128.7, 123.0, 122.7, 109.6, 108.4, 98.7, 87.5, 70.8, 70.3, 70.1, 69.7, 54.1, 45.3, 42.2, 40.3, 36.0, 34.9, 32.5, 32.4, 29.6, 26.6, 25.4. HRMS m/z (ESI) calcd for [$C_{44}H_{52}ClN_7O_7$] [M+H]$^+$: 826.3695, found: 826.3691. Purified by reverse phase HPLC using a gradient of 20-100% acetonitrile in water.

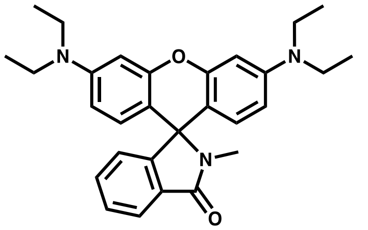

3',6'-bis(diethylamino)-2-methylspiro[isoindoline-1,9'-xanthen]-3-one (11). Synthesized according to general procedure for addition of nucleophiles into acyl chloride. 48 mg, 90% yield ^{1}H NMR (400 MHz, CDCl$_3$) δ 7.82 (dd, *J* = 6.3, 2.6 Hz, 1H), 7.32 (p, *J* = 5.5 Hz, 2H), 7.00 (dd, *J* = 5.9, 2.6 Hz, 1H), 6.37 (d, *J* = 8.8 Hz, 2H), 6.32 (d, *J* = 2.6 Hz, 2H), 6.20 (dd, *J* = 8.9, 2.6 Hz, 2H), 3.26 (q, *J* = 7.1 Hz, 8H), 2.59 (s, 3H), 1.09 (t, *J* = 7.0 Hz, 12H). ^{13}C{^{1}H} NMR (101 MHz, CDCl$_3$) δ 167.9, 153.8, 153.4, 148.7, 132.1, 130.9, 128.3, 127.9, 123.6, 122.8, 108.1, 105.4, 97.8, 64.6, 44.4, 24.8, 12.6. HRMS m/z (ESI) calcd for [$C_{29}H_{33}N_3O_2$] [M+H]$^+$: 456.2651,

found: 456.2642. Purified by normal phase flash chromatrography on 8 g RediSep® neutral silica using a gradient of 1-10% MeOH in DCM.

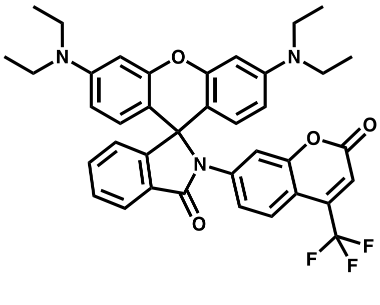

3',6'-bis(diethylamino)-2-(2-oxo-4-(trifluoromethyl)-2*H*-chromen-7-yl)spiro[isoindoline-1,9'-xanthen]-3-one (12). Synthesized according to general procedure for addition of nucleophiles into acyl chloride. 84 mg, 61% yield. ^{1}H NMR (400 MHz, $CDCl_3$) δ 7.91 (d, *J* = 7.1 Hz, 1H), 7.46 – 7.33 (m, 4H), 7.22 – 7.16 (m, 1H), 7.01 (d, *J* = 7.2 Hz, 1H), 6.55 (s, 1H), 6.51 (d, *J* = 8.8 Hz, 2H), 6.29 (d, *J* = 2.6 Hz, 2H), 6.18 (dd, *J* = 9.0, 2.6 Hz, 2H), 3.24 (q, *J* = 7.1 Hz, 8H), 1.08 (t, *J* = 7.0 Hz, 12H). ^{13}C{^{1}H} NMR (101 MHz, $CDCl_3$) δ 168.6, 159.3, 154.4, 154.1, 152.6, 149.0, 142.3, 141.3 (q, *J* = 32.8 Hz), 133.8, 128.6, 128.3, 128.0, 125.1 (q, *J* = 2.5 Hz), 123.8, 123.6, 122.8, 121.1, 120.1, 114.5 (q, *J* = 5.8 Hz), 112.4, 110.5, 108.3, 105.7, 98.0, 67.5, 44.3, 12.6. HRMS m/z (ESI) calcd for [$C_{38}H_{34}F_3N_3O_4$] [M+H]$^+$: 654.2580, found: 654.2570. Purified by normal phase flash chromatrography on 8 g RediSep® neutral silica using a gradient of 1-10% MeOH in DCM.

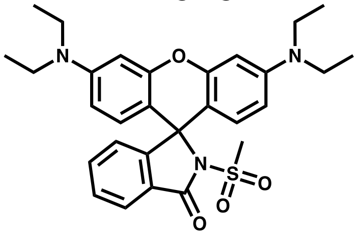

3',6'-bis(diethylamino)-2-(methylsulfonyl)spiro[isoindoline-1,9'-xanthen]-3-one (13). Synthesized according to general procedure for addition of nucleophiles into acyl chloride. 55 mg, 94% yield. ^{1}H NMR (400 MHz, $CDCl_3$) δ 7.90 (d, *J* = 7.5 Hz, 1H), 7.50 (t, *J* = 7.5 Hz, 1H), 7.43 (t, *J* = 7.4 Hz, 1H), 7.04 (d, *J* = 7.7 Hz, 1H), 6.46 (d, *J* = 8.8 Hz, 2H), 6.34 (d, *J* = 2.7 Hz, 2H), 6.21 (dd, *J* = 8.9, 2.6 Hz, 2H), 3.26 (q, *J* = 7.1 Hz, 8H), 2.85 (s, 3H), 1.09 (t, *J* = 7.0 Hz, 12H). ^{13}C{^{1}H} NMR (101 MHz, $CDCl_3$) δ 167.5, 153.6, 153.4, 149.1, 135.1, 128.9, 128.0, 124.7, 123.9, 107.6, 105.7, 97.8, 44.3, 41.9, 12.6. HRMS m/z (ESI) calcd for [$C_{29}H_{33}N_3O_4S$] [M+H]$^+$: 520.2270, found: 520.2265. Purified by normal phase flash chromatrography on 8 g RediSep® neutral silica using a gradient of 1-10% MeOH in DCM.

4. **Analytical Data**

Compound 1b

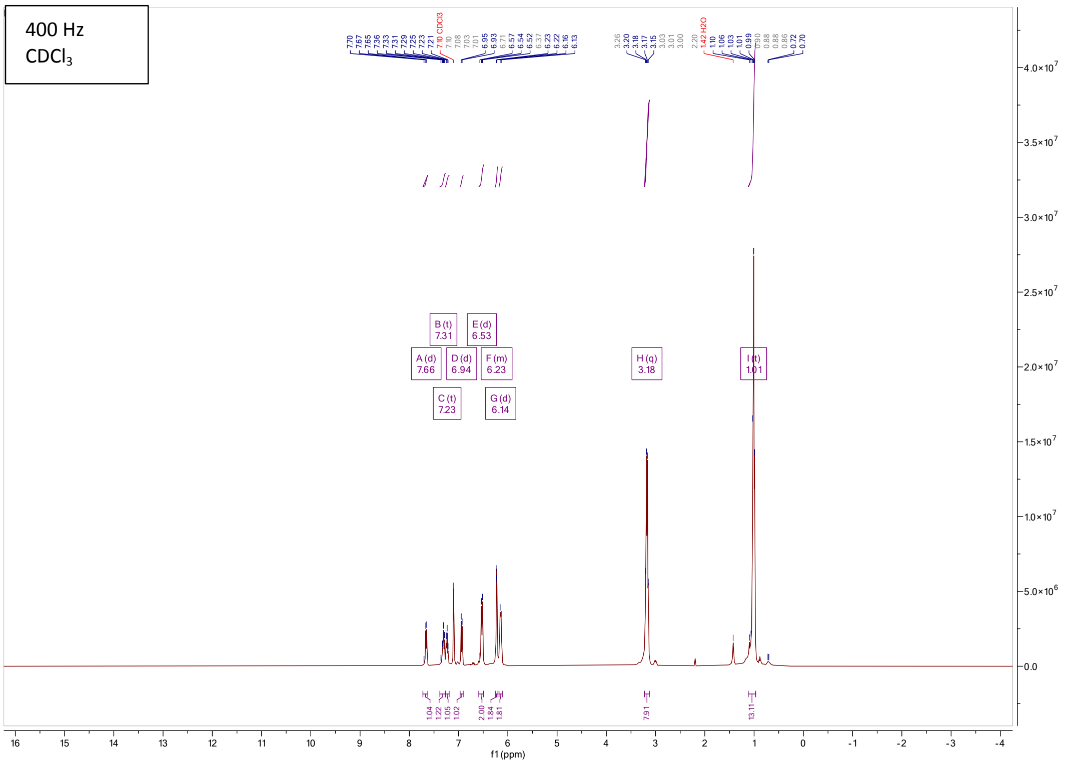
400 Hz
CDCl3

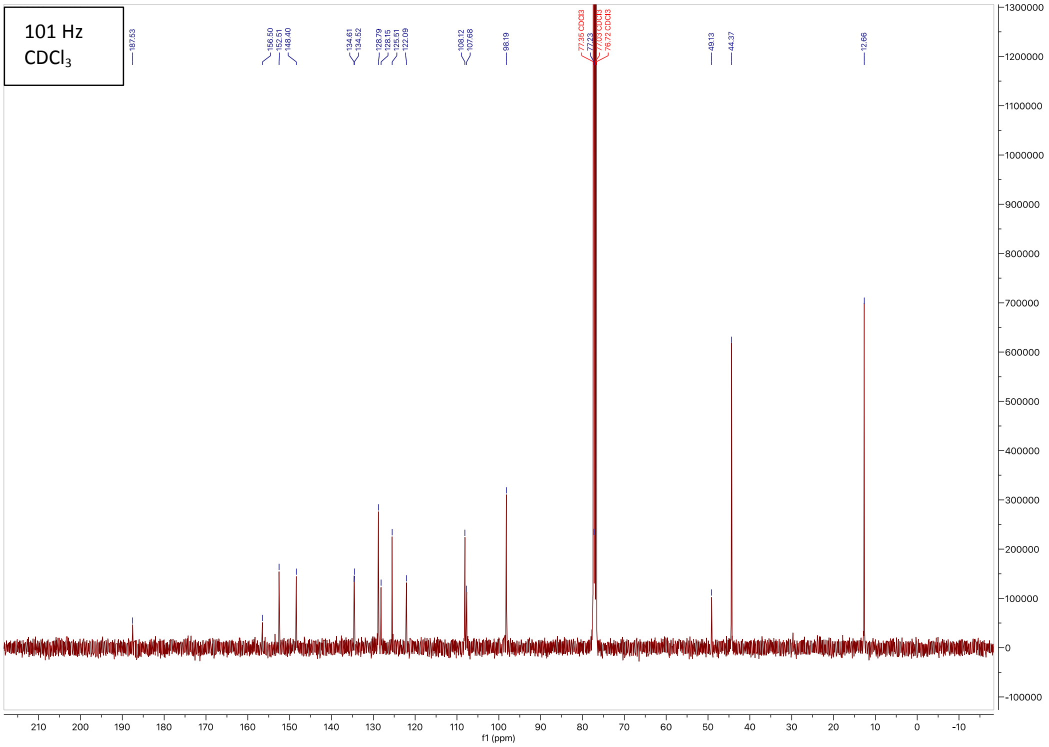
101 Hz
CDCl3

Compound 2b

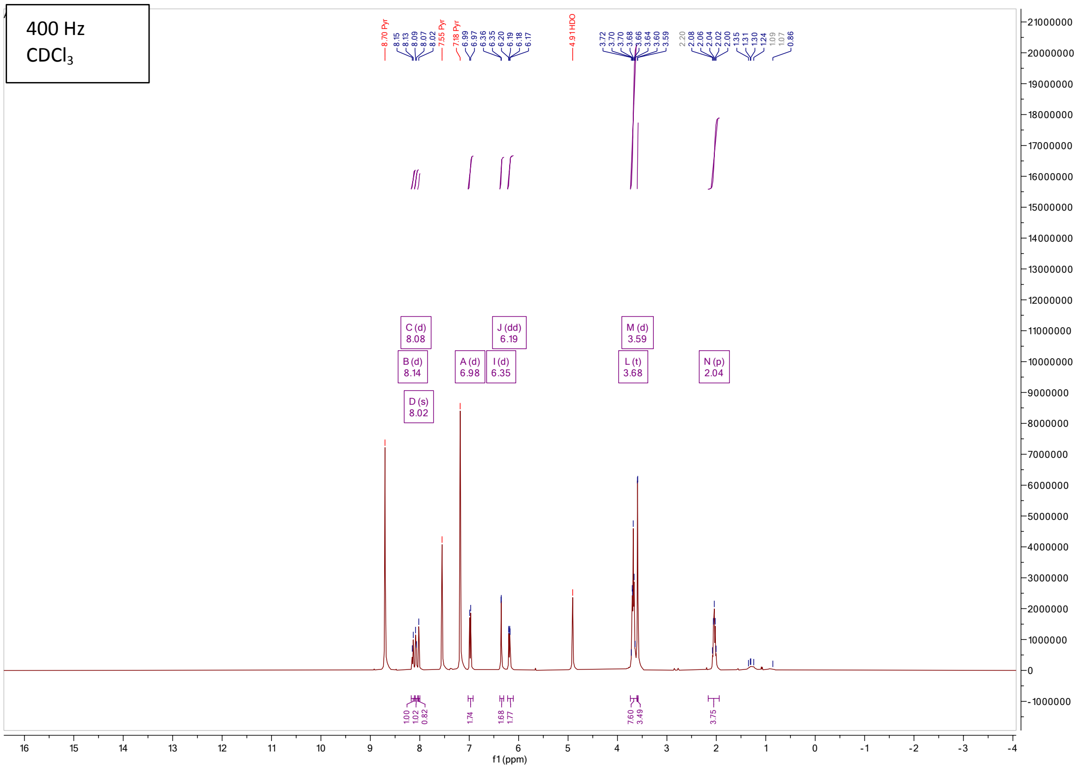

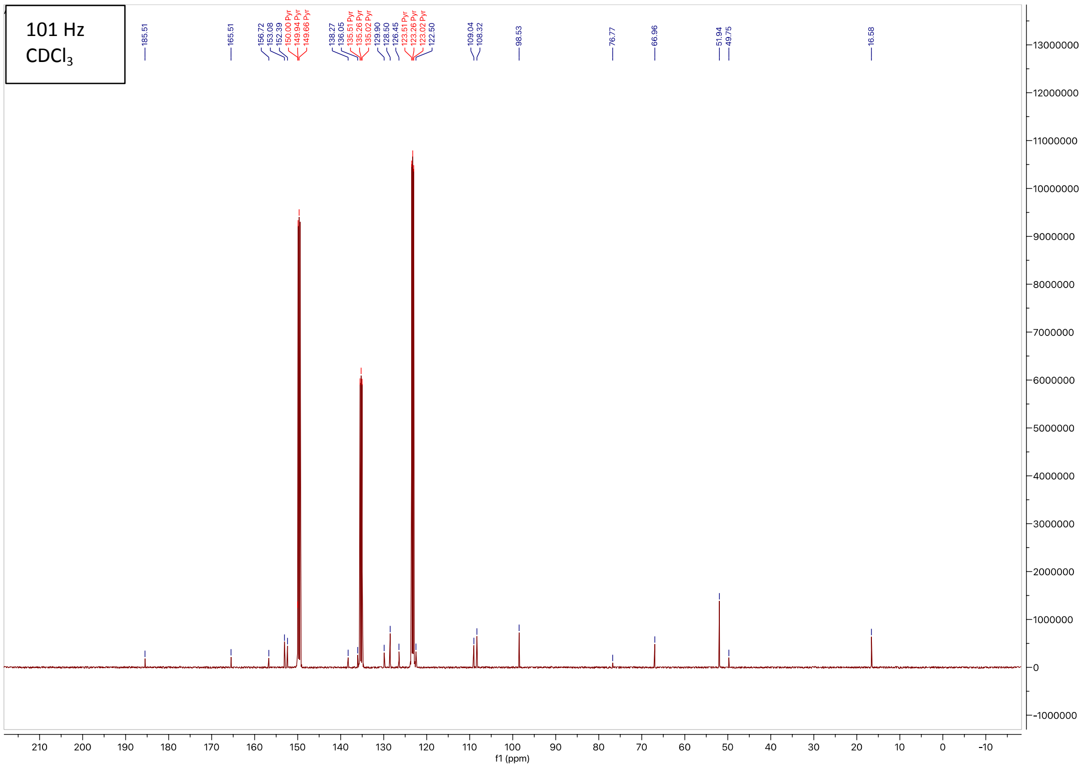

Compound 3b

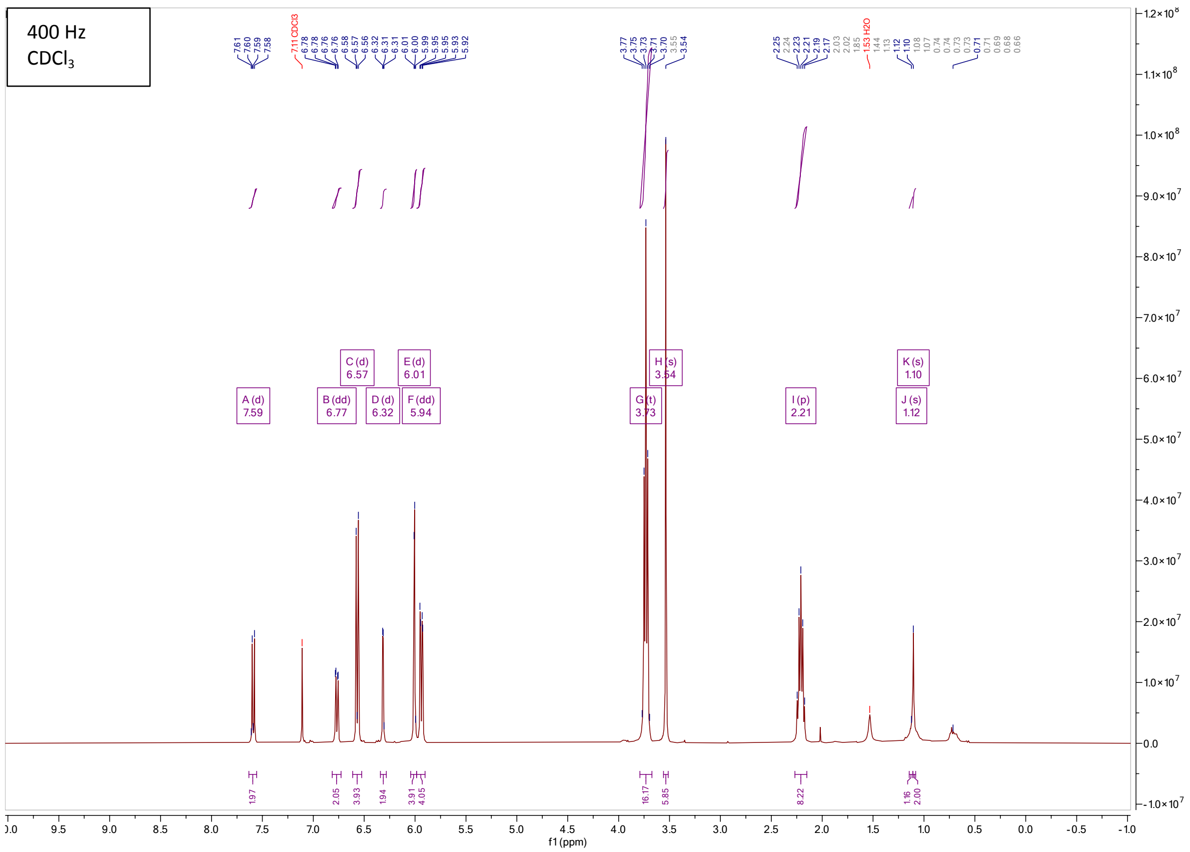

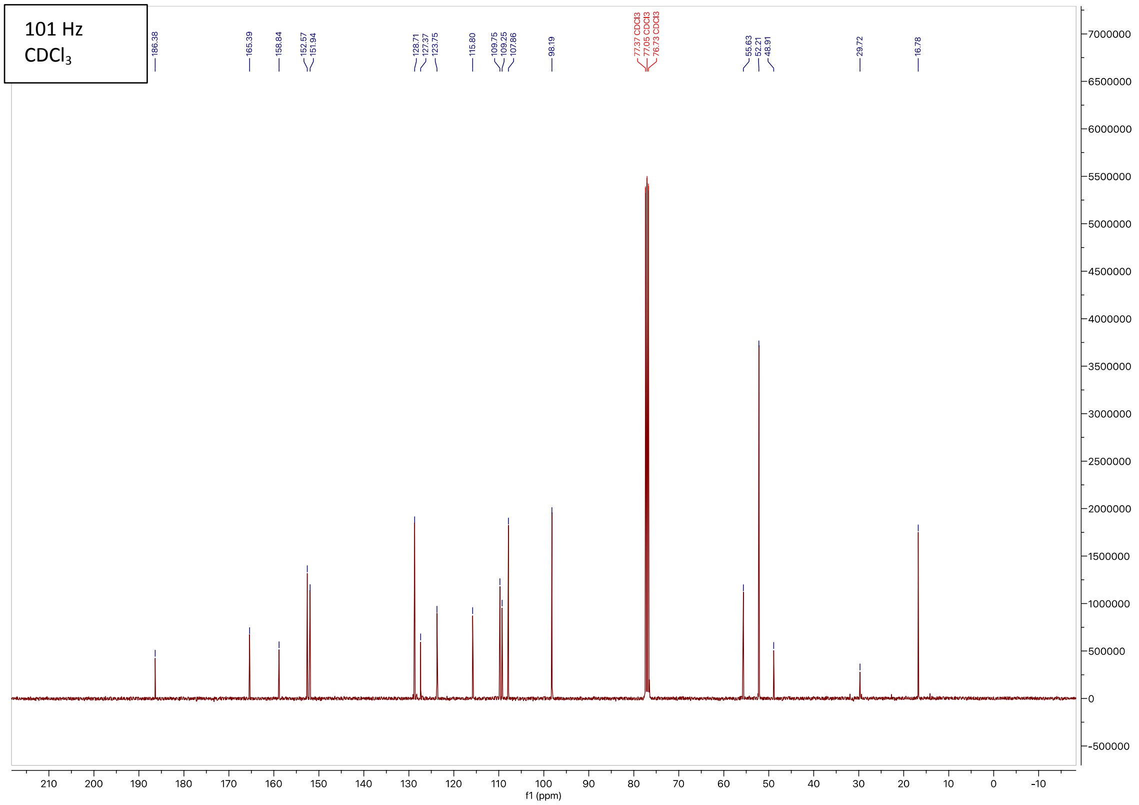

400 Hz
Pyridine-d5

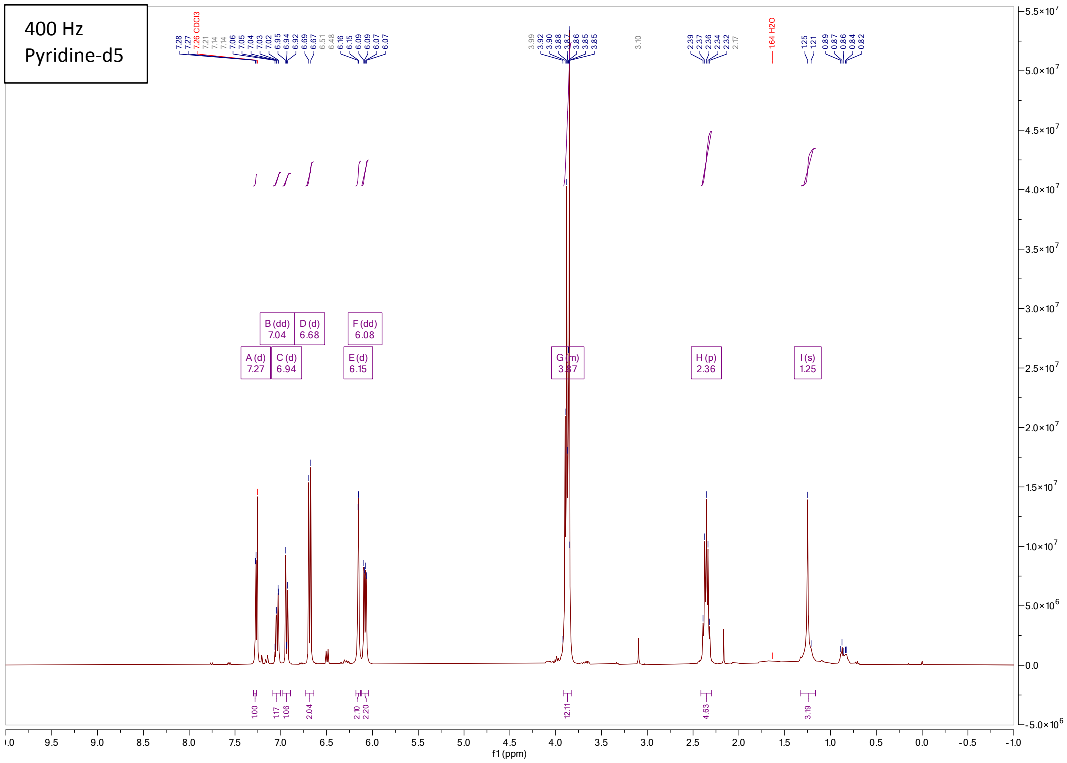

Compound 4b

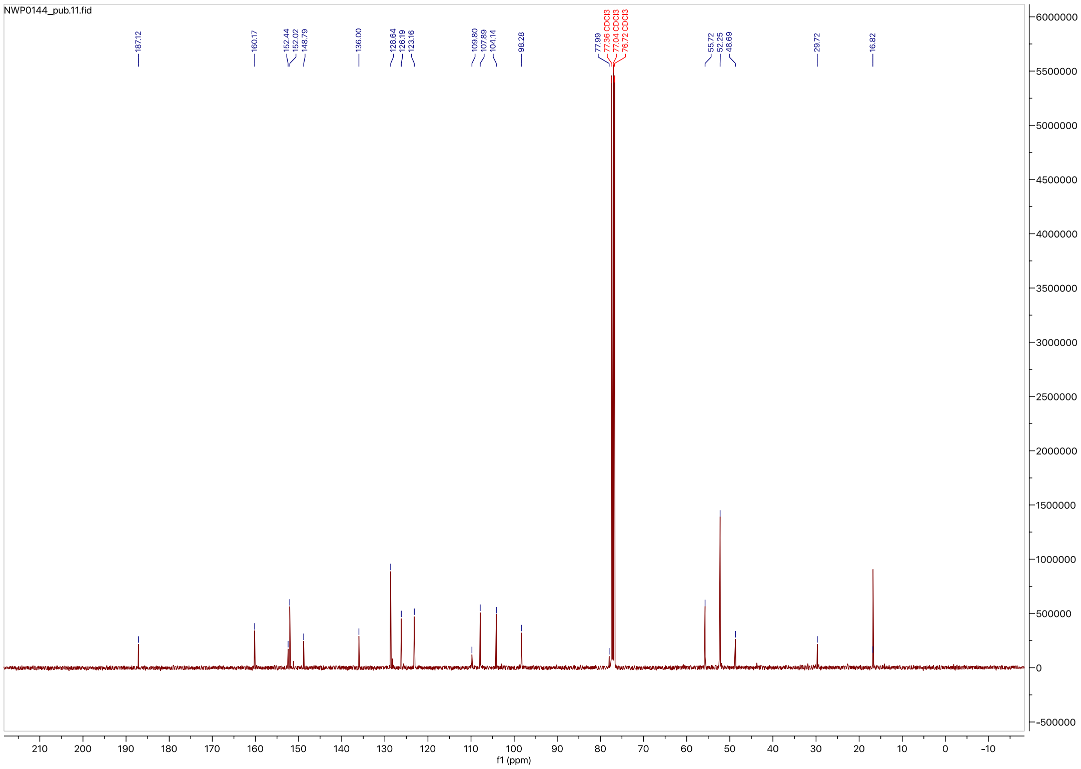

101 Hz
Pyridine-d5

Compound 5b

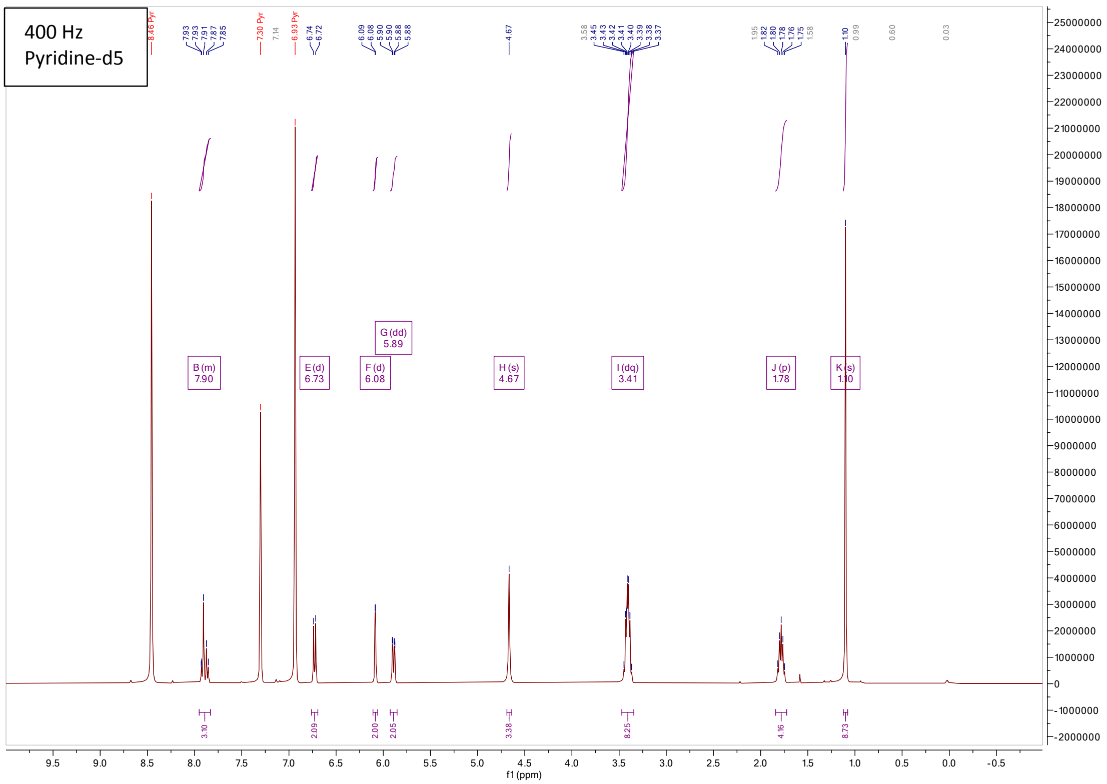

400 Hz
Pyridine-d5
B (m) 7.90
E (d) 6.73
F (d) 6.08
G (dd) 5.89
H (s) 4.67
I (dq) 3.41
J (p) 1.78
K (s) 1.10
f1 (ppm)

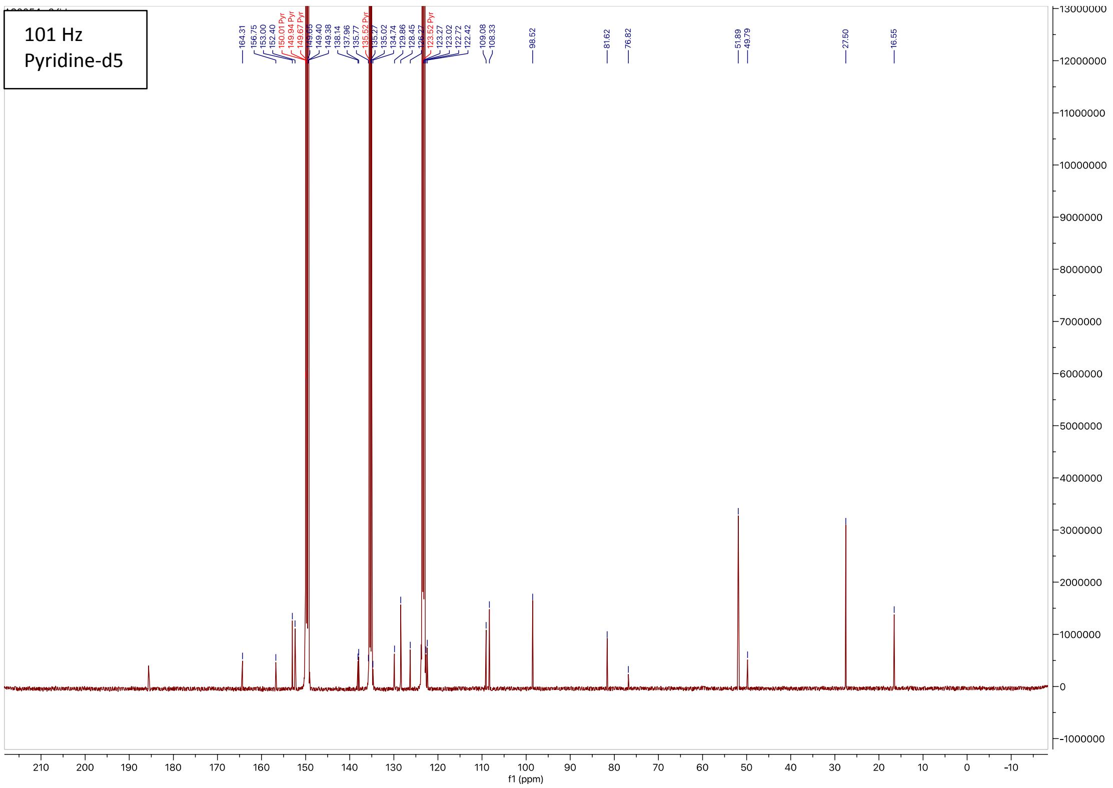

101 Hz
Pyridine-d5
f1 (ppm)

COMPOUND 6B

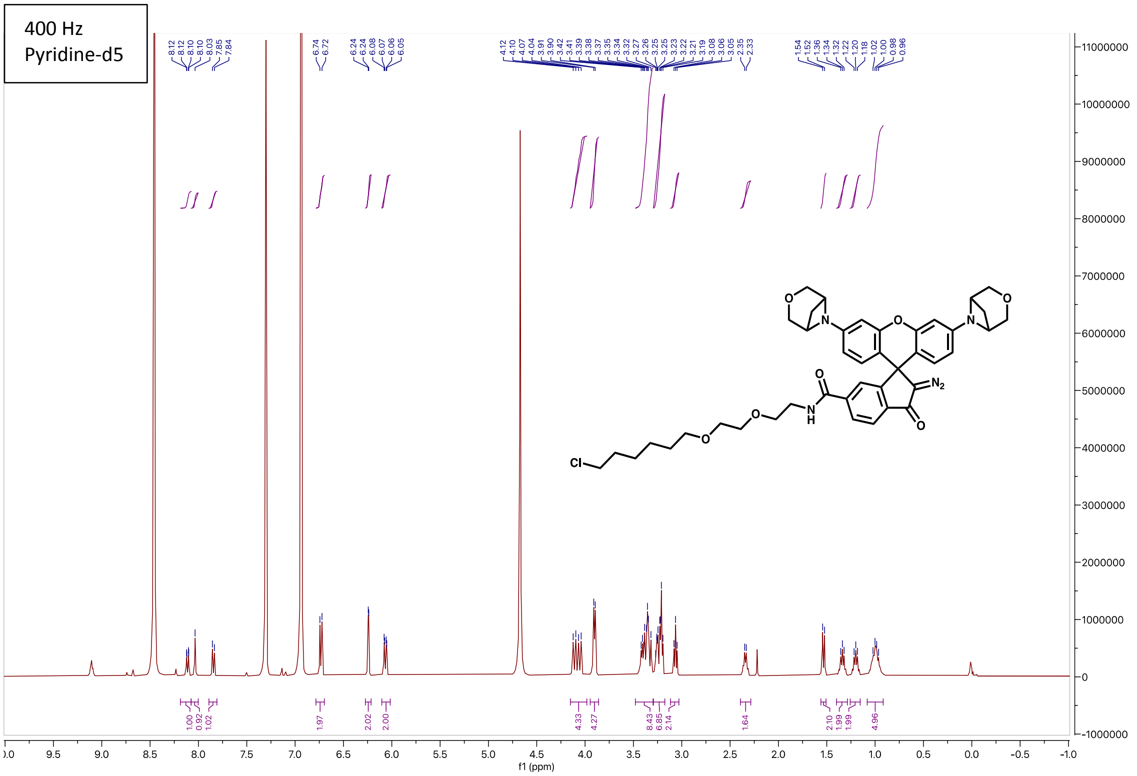

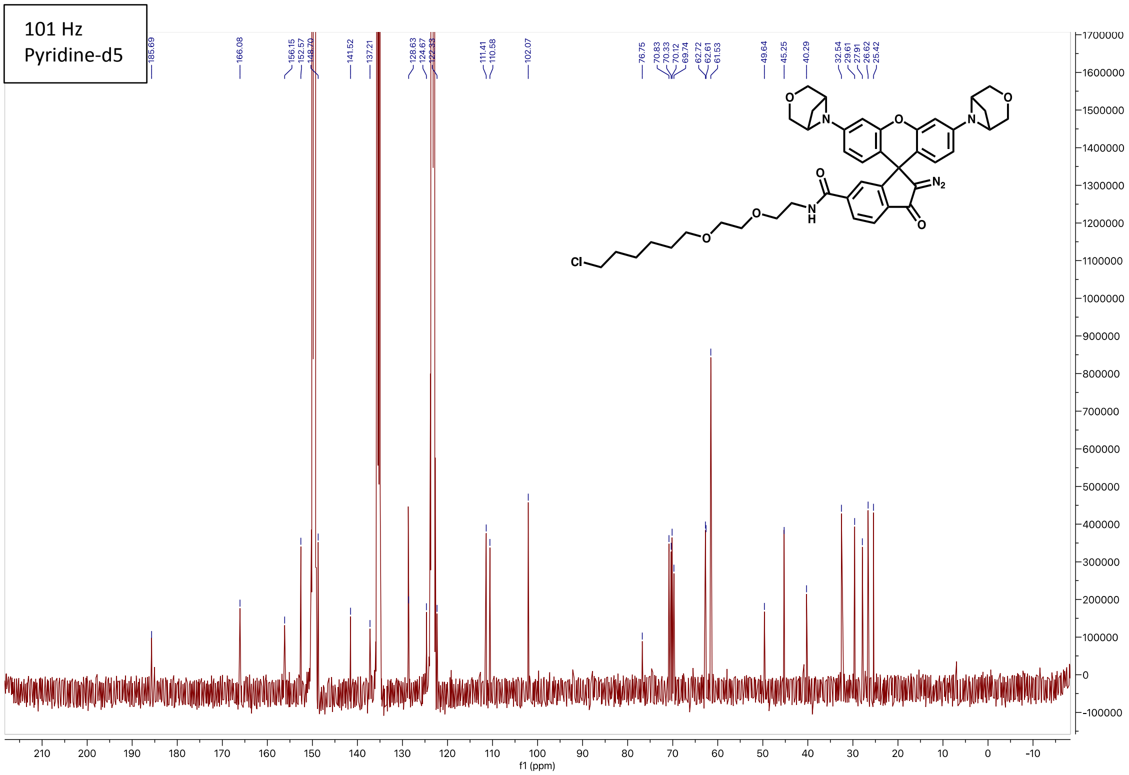

Compound 7b

Mobile phase-A: 0.1%Formic Acid In Water.Mobile phase-B: 100% ACN.
Column – XBRIDGE C18 3.5μm, 4.6X50mm
Flow-1mL/min, Temp: 40°C
Time (min) and %B: 0-5; 0.3-5; 2-95; 4-95; 4.5-5;6-5.

ANL-BLR-LCMS-015 22-Jul-2022 20:14:29
ARG-EKT-1007-548A1

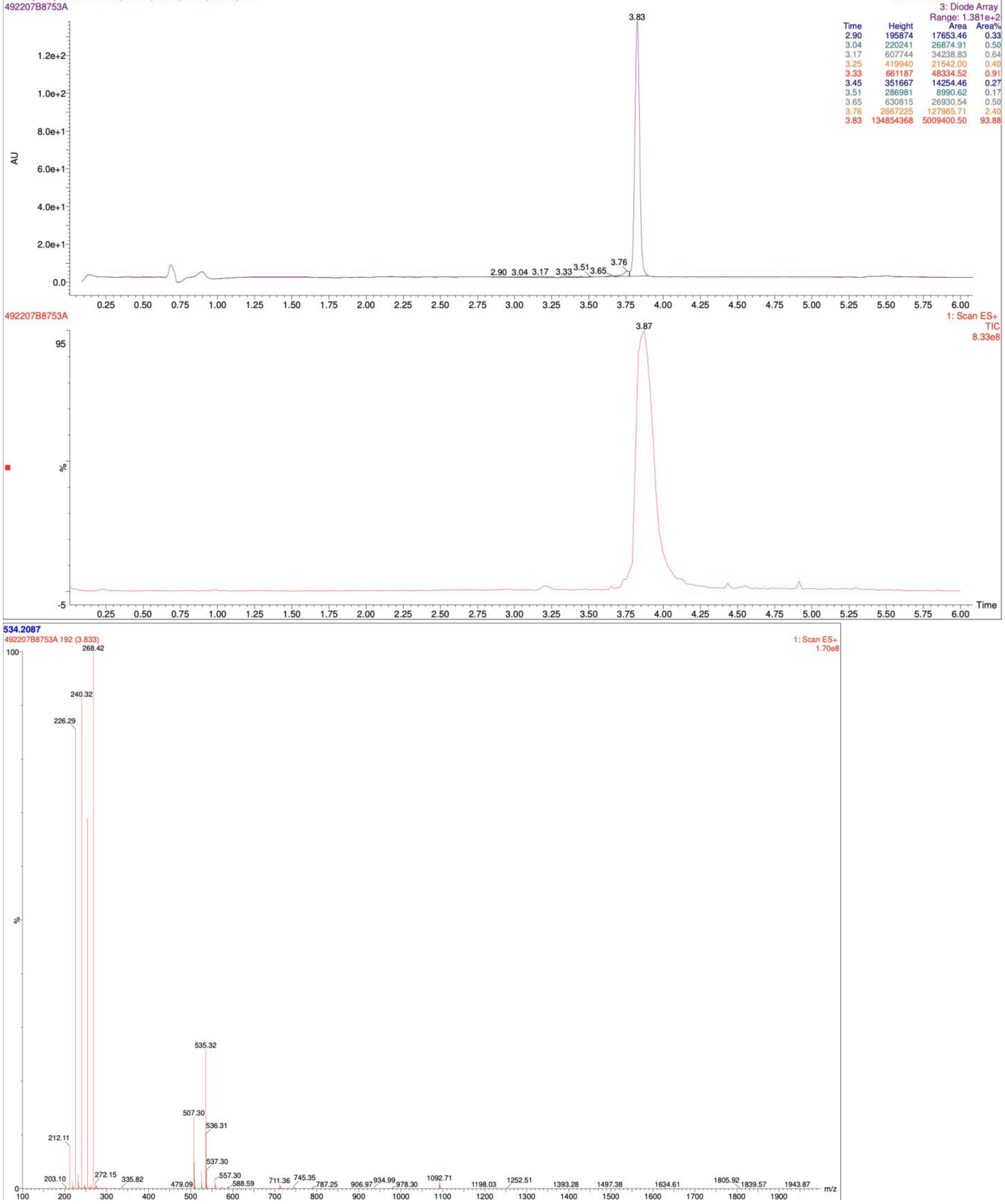

Time	Height	Area	Area%
2.90	195874	17653.46	0.33
3.04	220241	26874.91	0.50
3.17	607744	34238.83	0.64
3.25	419940	21542.00	0.40
3.33	661187	48334.52	0.91
3.45	351667	14254.46	0.27
3.51	286981	8990.62	0.17
3.65	630815	26930.54	0.50
3.76	2667225	127985.71	2.40
3.83	134854368	5009400.50	93.88

Compound 8b

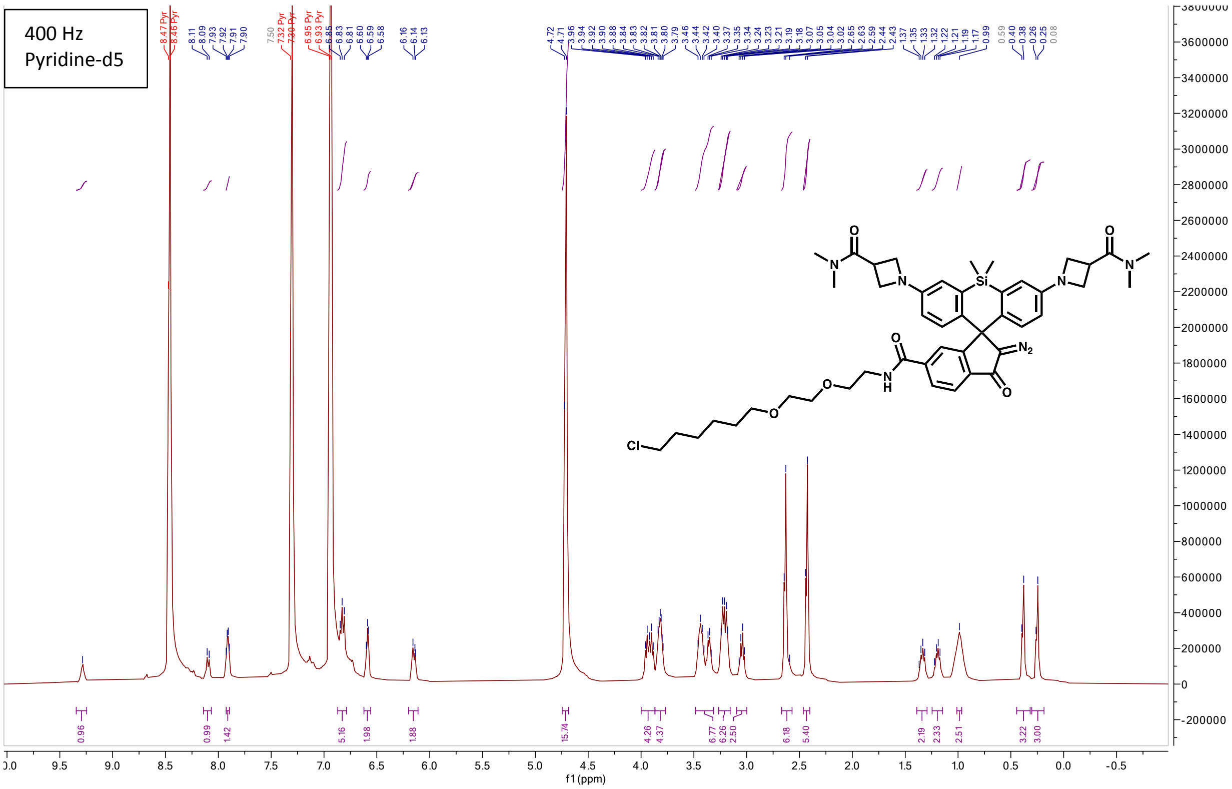

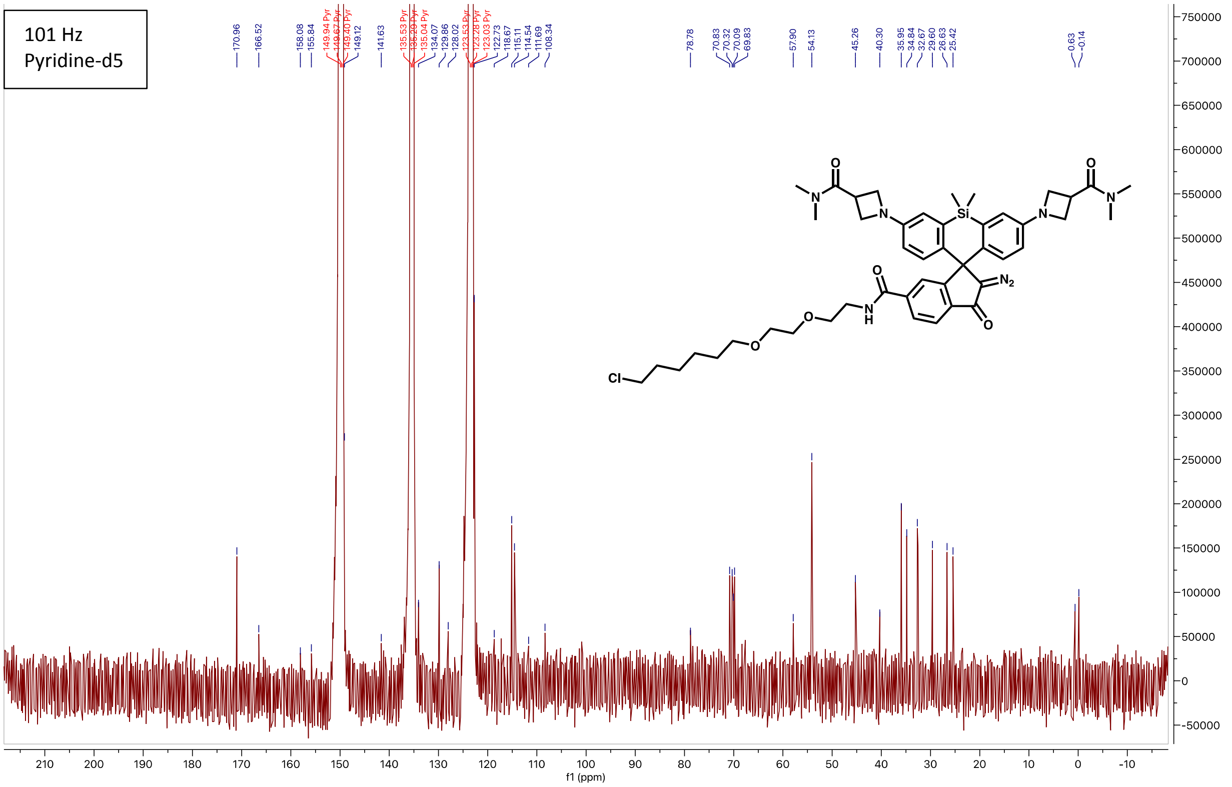

Compound 10b

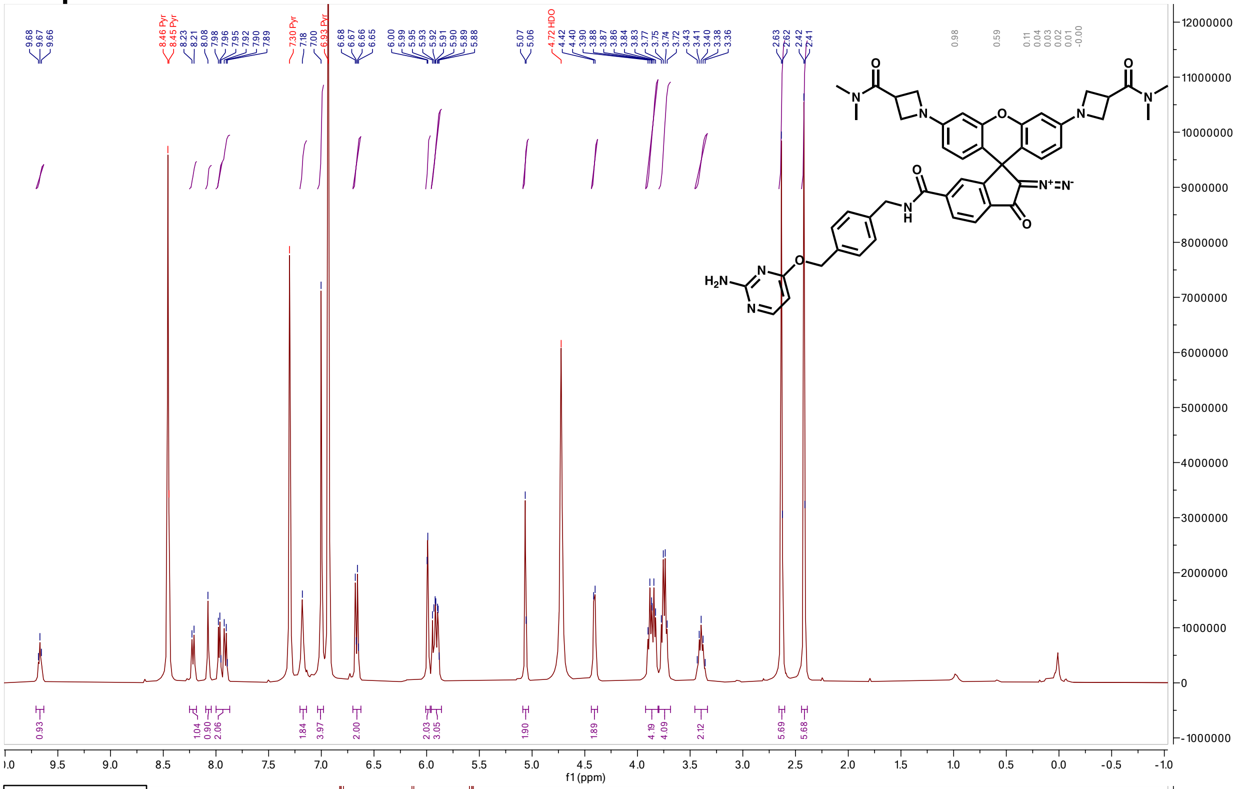

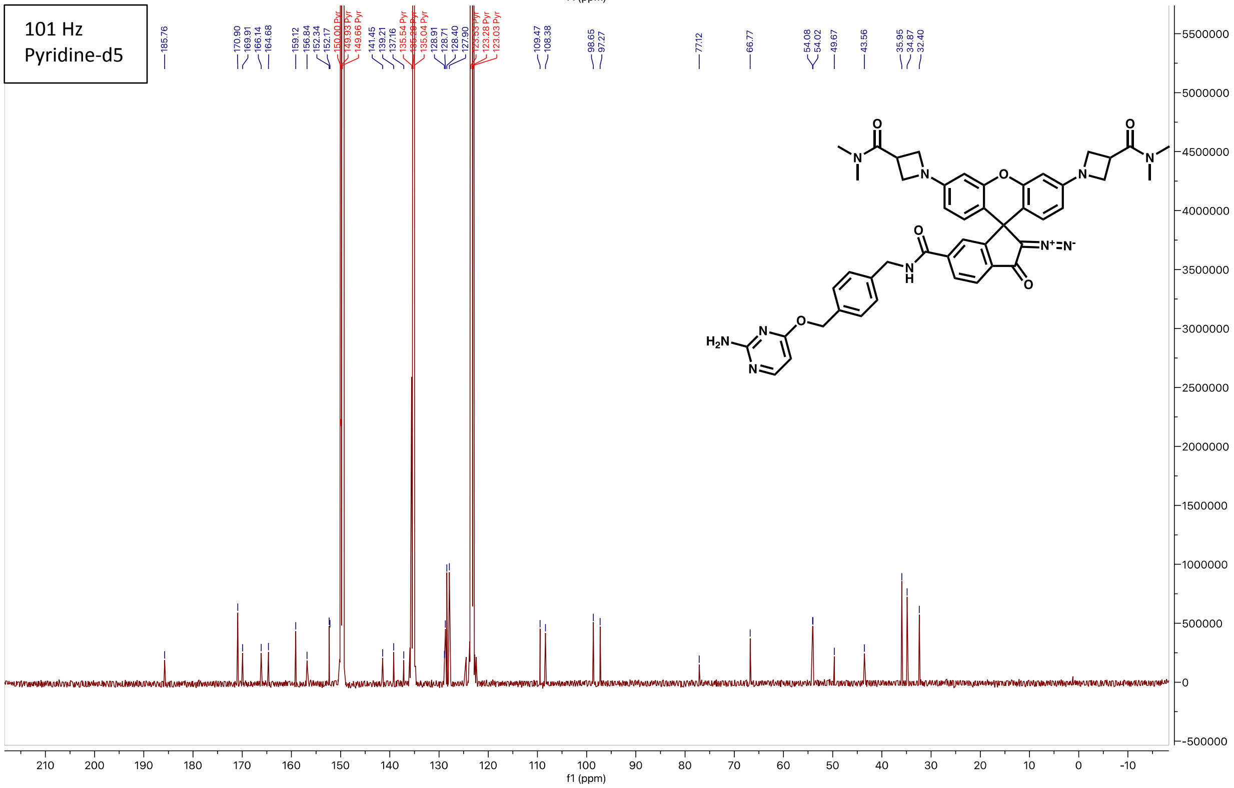

Compound 10c

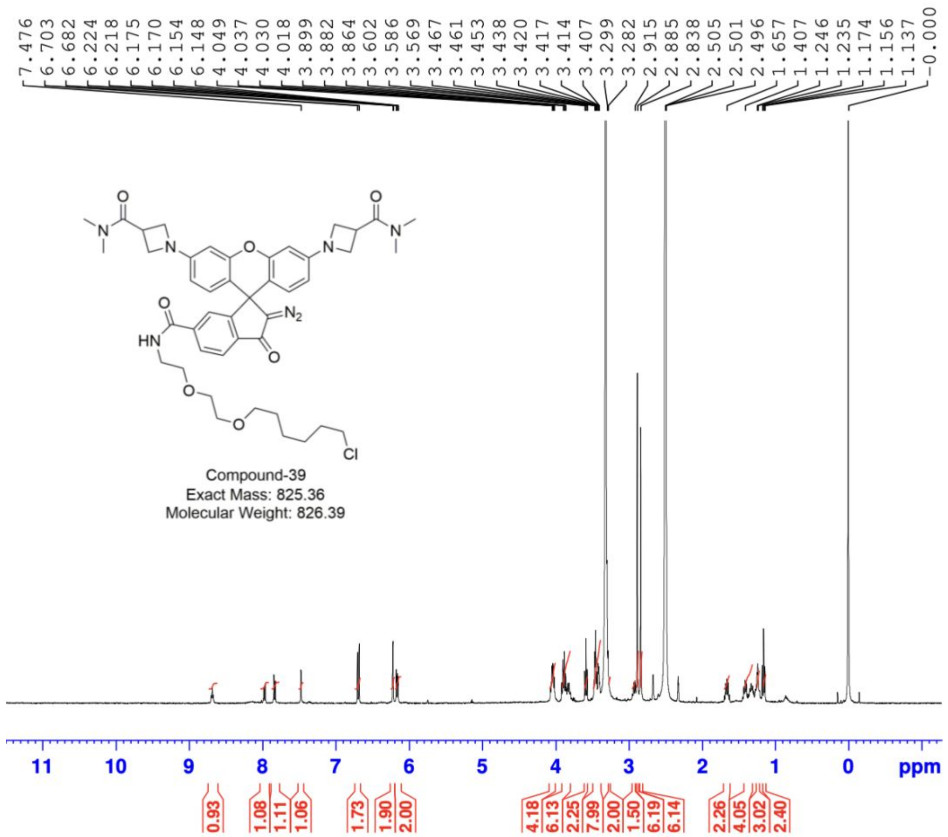

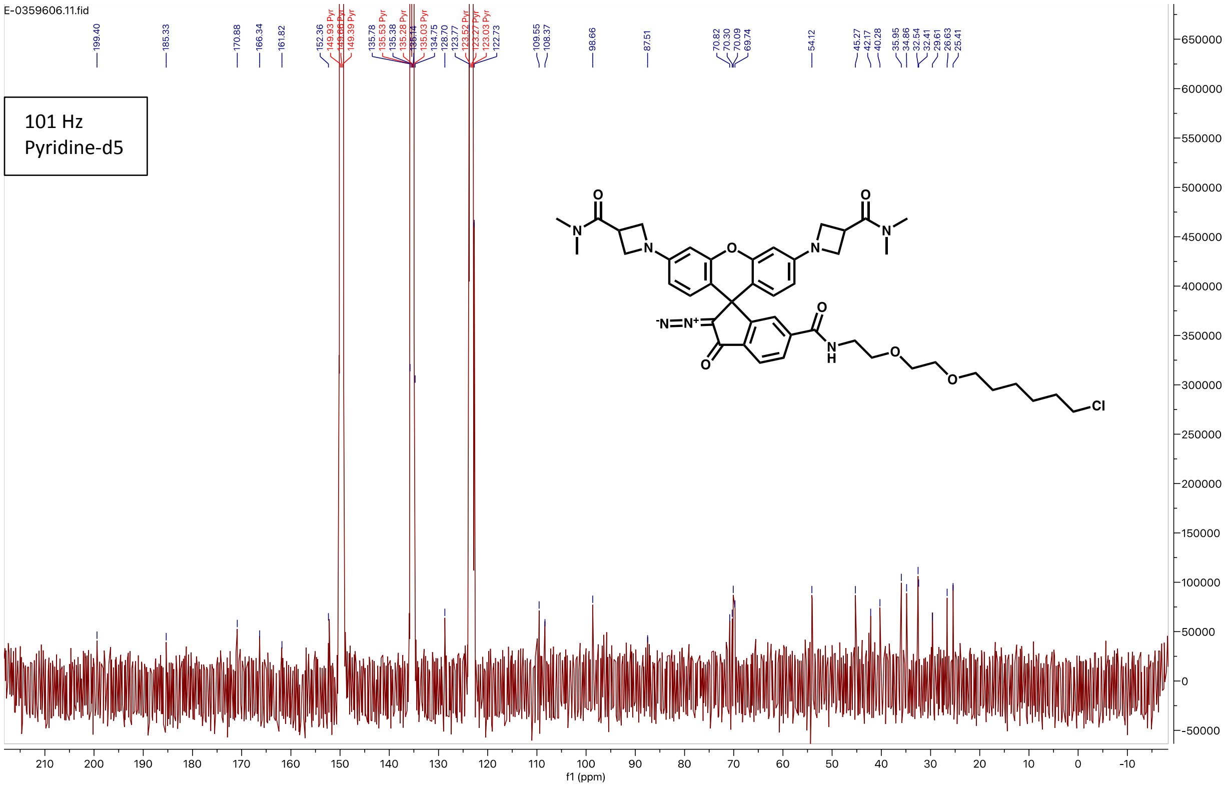

Compound 11

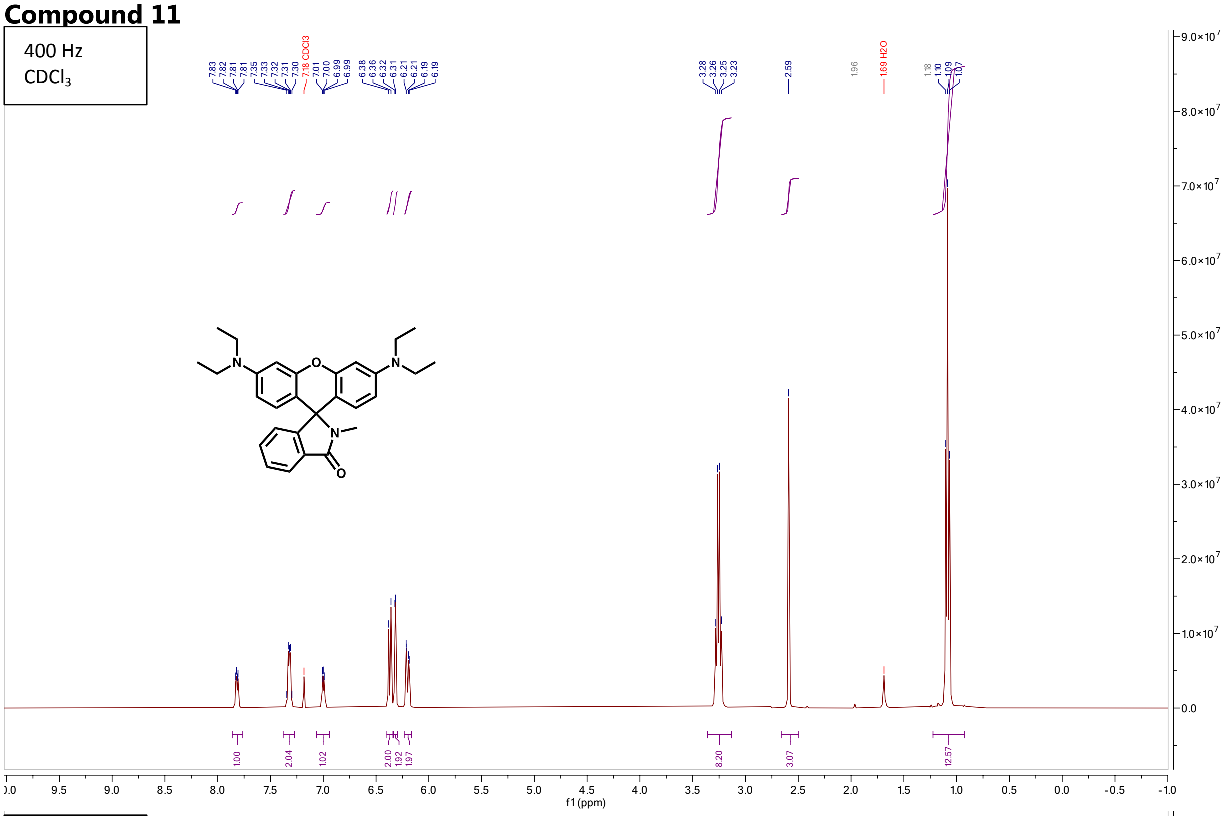
400 Hz
$CDCl_3$
7.83 7.82 7.81 7.81 7.35 7.33 7.32 7.31 7.30
7.18 CDCl3
7.01 7.00 6.99 6.99 6.38 6.36 6.32 6.31 6.21 6.21 6.19 6.19
3.28 3.26 3.25 3.23
2.59
1.96
1.69 H2O
1.18 1.10 1.09 1.07
1.00 2.04 1.02 2.00 1.92 1.97 8.20 3.07 12.57
f1 (ppm)

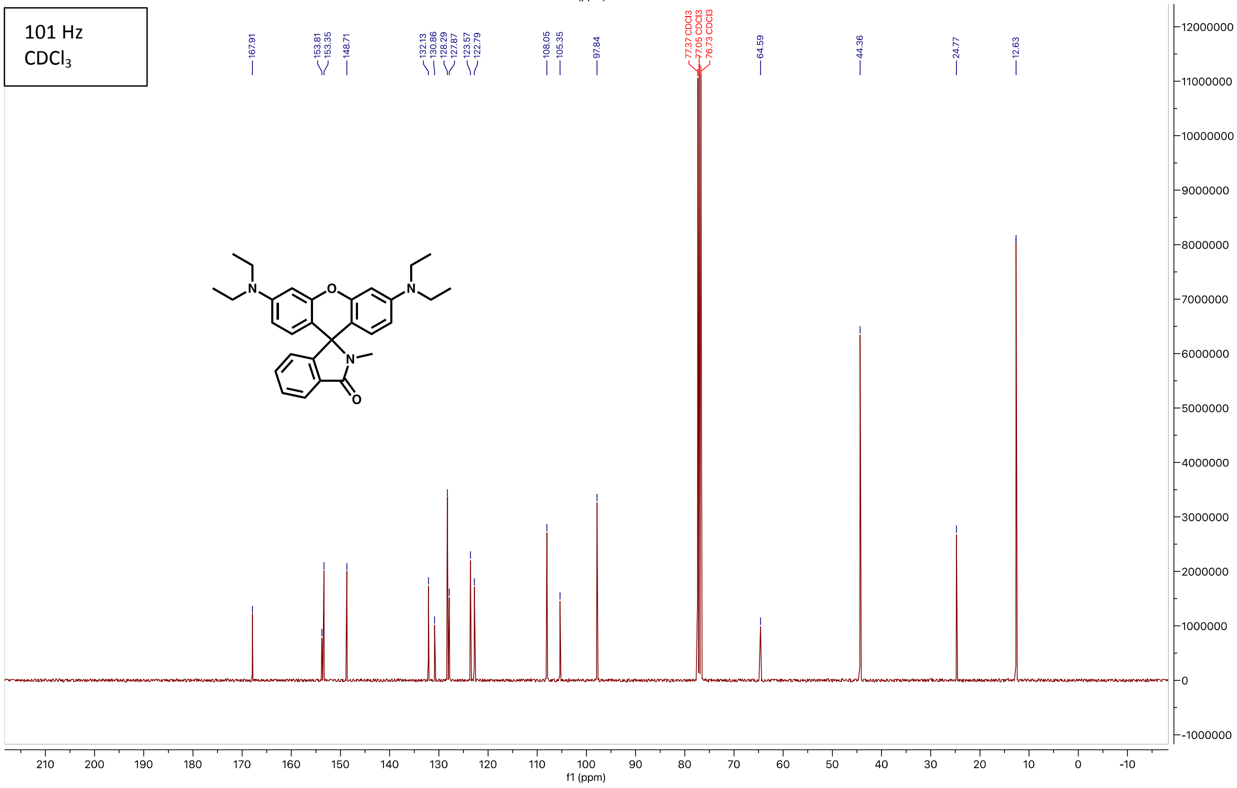
101 Hz
$CDCl_3$
167.91
153.81 153.35 148.71
132.13 130.86 128.29 127.87 123.57 122.79
108.05 105.35 97.84
77.37 CDCl3 77.05 CDCl3 76.73 CDCl3
64.59
44.36
24.77
12.63
f1 (ppm)

Compound 12

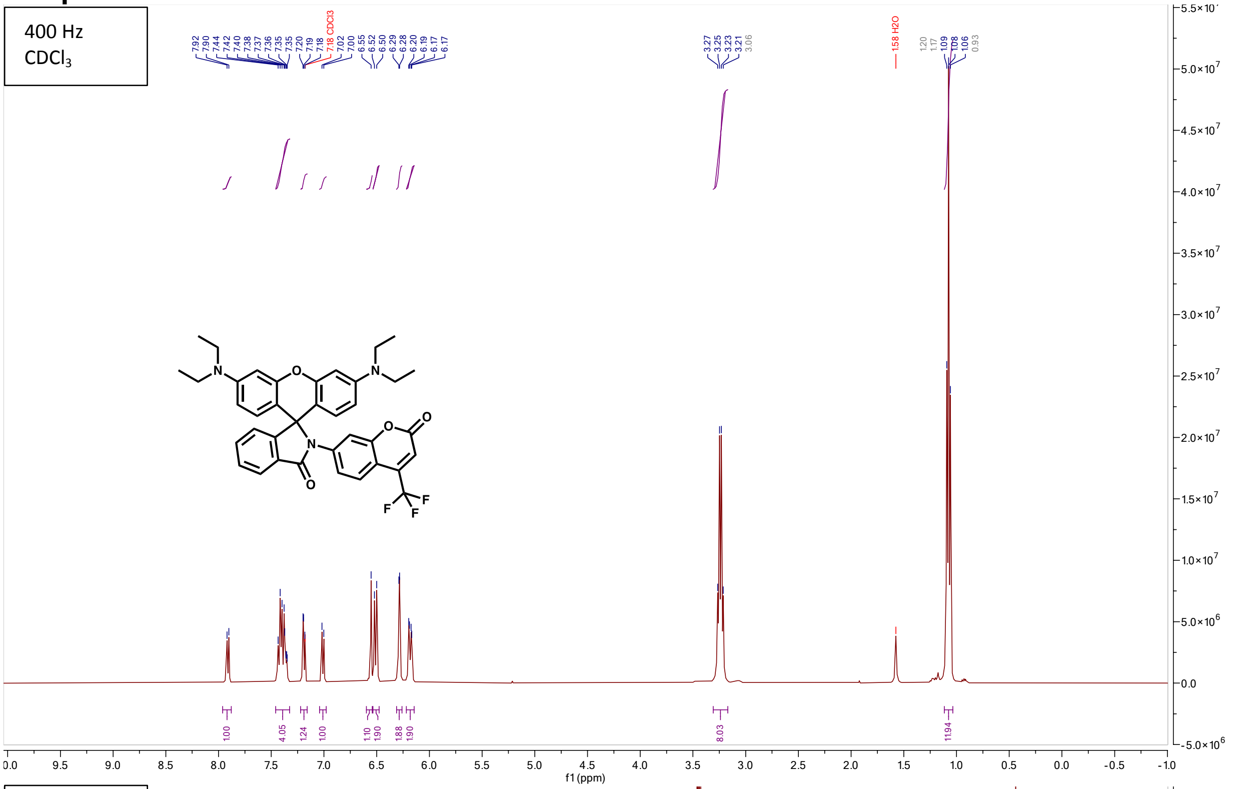

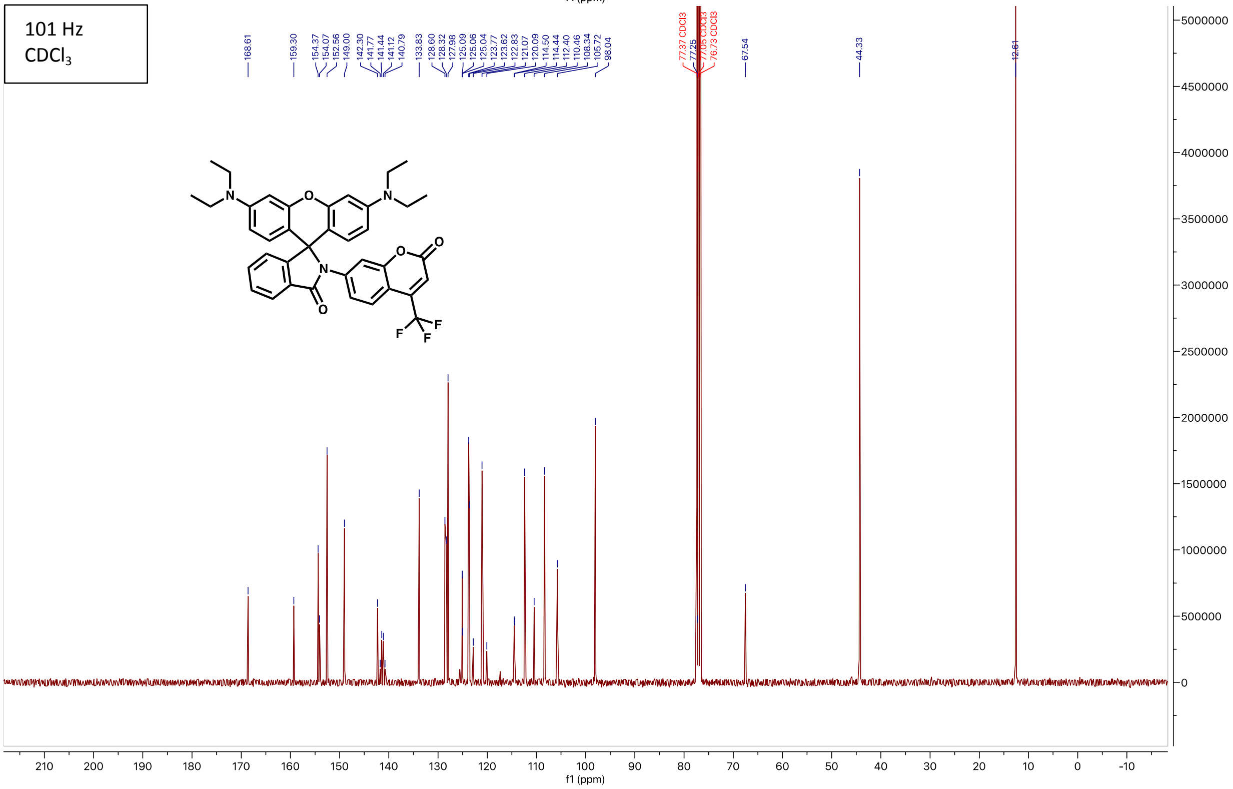

Compound 13

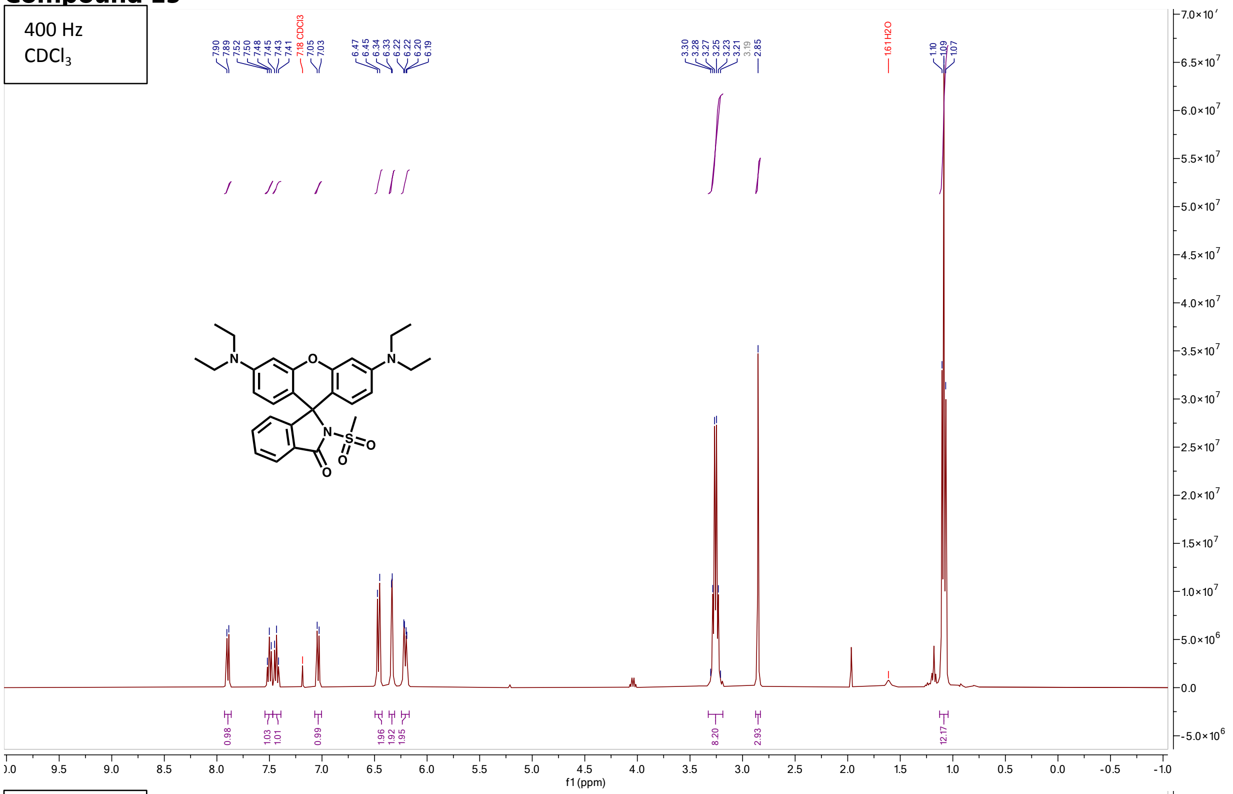

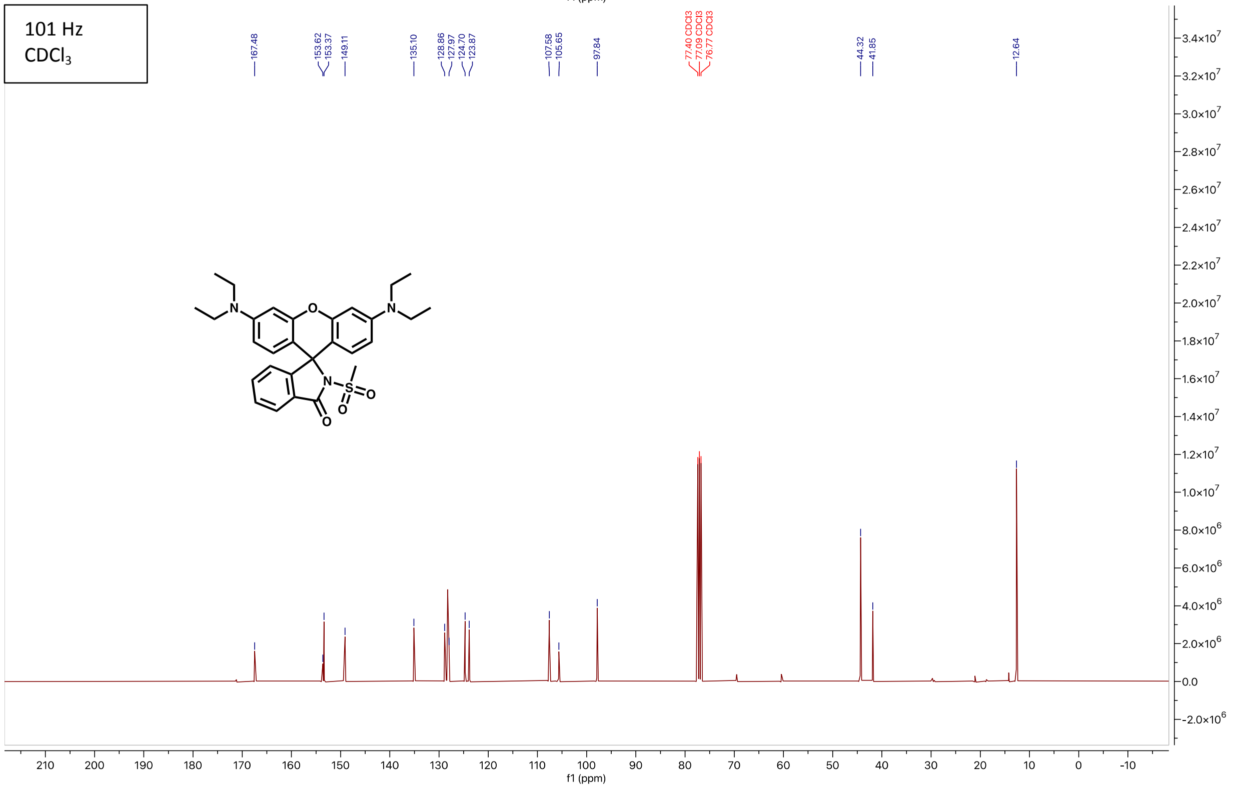

3. SINGLE MOLECULE TRACKING SAMPLE PREPARATION

U2OS cells (HBT-96, ATCC), or U2OS cells bearing an endogenous HaloTag knock-in were seed on tissue culture treated 384-well glass-bottom plates at 6000 cells per well. Seeded cells were then incubated at 37 °C and 5% CO_2 to allow adhesion overnight. For all SMT experiments, cells were incubated with 50 nM of Compound 1 or PA-JF_{549}, or 50 pM JF_{549} (Cat. No. GA1110, Promega) for an hour in complete medium. Cells were then washed three times in DPBS and twice in imaging media, which is fluoroBrite DMEM media (Cat. No. A1896701, Thermo Fisher) supplemented with GlutaMAX (Cat. No. 35050079, Thermo Fisher) and the same serum and antibiotics as growth media.

4. SINGLE MOLECULE TRACKING IMAGE ACQUISITION

Image acquisition using SMT was performed on a custom-built HILO microscope described in McSwiggen et al[6] based on a Nikon Ti2, motorized stage, stage top environmental chamber (OKO labs), quad-band filter cube (Chroma), custom laser launch with 405 nm, and 561 nm wavelengths, delivering 0 - 10 mW and >150 mW of power to the back focal plane of the objective, respectively. Fluorescence emission was passed through a high-speed filter wheel (Finger Lakes Instruments) and collected with a backlit CMOS camera (Prime 95b, Teledyne). Images were acquired with a 60X 1.27 NA water immersion objective (Nikon). Environmental chamber was set to 37° Celsius, 95% humidity, and 5% CO_2. 5000 frames of images were collected with step-wise increases in 405 illumination intensity to account for depletion of the un-imaged pool of molecules over time.

5. SINGLE MOLECULE TRACKING IMAGE ANALYSIS

Tracking proceeded as described in McSwiggen et al,[6] but is described here in brief. Individual SMT movies were processed in three sequential steps – detection, subpixel localization, and linking – using a combination of existing methods. Briefly, spots were detected using a generalized log likelihood ratio detector. After detection, the estimated position of each emitter was refined to subpixel resolution using Levenberg-Marquardt fitting with an integrated 2D Gaussian spot model starting from an initial guess afforded by the radial symmetry method. Detected spots were linked into trajectories using a custom modification of a hill-climbing algorithm. The results are a table of spot coordinates with an estimate of their diffusion coefficient. To visualize dynamical states as a function of their position in the image, spots falling within a defined range of diffusion coefficients were rendered into an image reconstruction with the same pixel dimensions as the original image.

To recover dynamical information from trajectories, we used state arrays (Heckert), a Bayesian inference approach, with the "RBME" likelihood function and a grid of 125 diffusion coefficients from 0.003 to 30 $\mu m^2 s^{-1}$ and 7 localization error magnitudes from 0.02 to 0.08 µm. After inference, localization error was marginalized out to yield a one-dimensional distribution over the diffusion coefficient for each field of view.

6. EXTENDED OPTIMIZATION DATA

Table S1 Optimization of carboxylic acid activation and subsequent diazoketone synthesis.

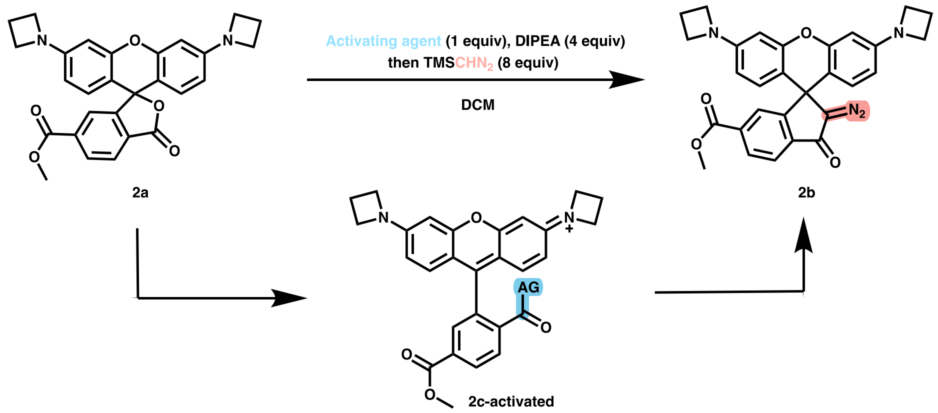

Entry[a]	Activating agent	2a (%)[b]	2b (%)[b]	2c (%)[bd]
1	Ghosez' reagent	0	53[e]	47
2	$(COCl)_2$	50	50	0
3	Boc anhydride	100	0	0
4	EtOCOCl	100	0	0
5	iBuOCOCl	47	0	53
6	HATU	75	0	25
7	PyBOP	68	12[f]	20
8	PyClop	65	0	35
9	PyBrop	58	0	42
10	T3P	100	0	0

[a]Reactions run on a 0.1 mmol scale at 0.05 M with respect to starting material. [b]Product distribution determined by LC-MS using percent of total area under the curve. [d]When AG = Cl, percent **2c** evaluated as the corresponding methyl amide after quenching the reaction mixture with methylamine. [e]Observed 50% HCl-opened azetidine as significant byproduct [f]**2b** only observed when heated to 40° C.

Table S2 Solvent optimization of acid chloride and subsequent diazoketone synthesis.

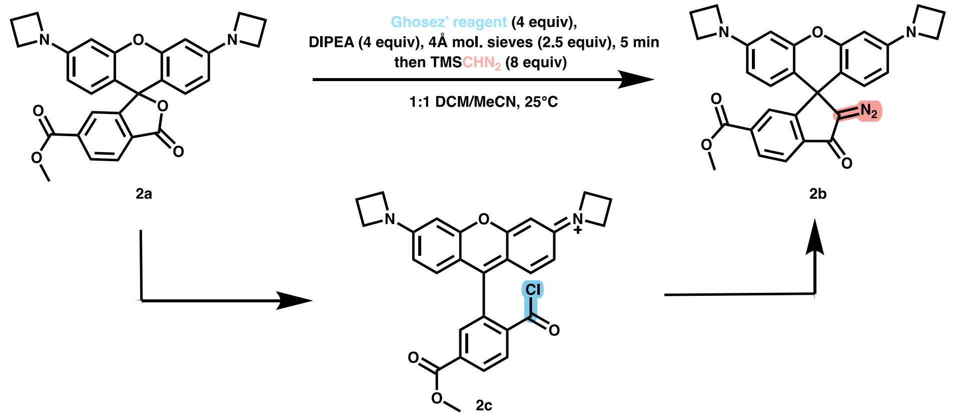

Entry[a]	Solvent	2a (%)[b]	2b (%)[b]	2c (%)[bd]
1	1:1 MeCN/DCM	7	91	2
2	1:2 MeCN/DCM	10	90	0
3	2:1 MeCN/DCM	5	90	5
4	THF	85	0	15
5	DCM	0	53[e]	47
6	$CHCl_3$	100	0	0
8	Dioxane	97	0	3
9	Toluene	89	0	11
10	MeCN	10	60	30

[a]Reactions run on a 0.1 mmol scale at 0.05 M with respect to starting material. [b]Product distribution determined by LC-MS using percent of total area under the curve. [d]Percent **2c** evaluated as the corresponding methyl amide after quenching the reaction mixture with methylamine. [e]Observed 50% HCl-opened azetidine as significant byproduct

7. SOLUBILITY AND PERMEABILITY DATA

Table S3 Solubility and permeability data for compounds featured in **Figure 1a.** Data indicate demonstrate that 10c has improved properties over photoactivatable JF549-HaloTag that are more similar to JF549-HaloTag. Data are taken

Compound	Solubility (μM)	Permeability (MDCK P_{app} A->B, x 10^{-6}, cm/s)
JF549-HaloTag	> 200	3.0
Photoactivatable JF549-HaloTag	1.3	< 0.04
Compound 10c	3.7	4
JF646-HaloTag	BLOQ[a]	< 0.01
Photoactivatable JF646-HaloTag	BLOQ[a]	< 0.01
Compound 8b	0.059	< 0.03
Compound 10b	0.098	< 0.04

to justify reduced non-specific labeling by 10c over photoactivatable JF5 49-HaloTag

[a]Below limit of quantification

8. REFERENCES

1 P. A. Bray and R. K. Sokas, *Journal of Occupational and Environmental Medicine*, 2015, **57**, e15.
2 V. N. Belov, C. A. Wurm, V. P. Boyarskiy, S. Jakobs and S. W. Hell, *Angewandte Chemie International Edition*, 2010, **49**, 3520–3523.
3 J. B. Grimm and L. D. Lavis, *Org. Lett.*, 2011, **13**, 6354–6357.

4 J. B. Grimm, B. P. English, J. Chen, J. P. Slaughter, Z. Zhang, A. Revyakin, R. Patel, J. J. Macklin, D. Normanno, R. H. Singer, T. Lionnet and L. D. Lavis, *Nat Methods*, 2015, **12**, 244–250.
5 J. B. Grimm, B. P. English, H. Choi, A. K. Muthusamy, B. P. Mehl, P. Dong, T. A. Brown, J. Lippincott-Schwartz, Z. Liu, T. Lionnet and L. D. Lavis, *Nat Methods*, 2016, **13**, 985–988.
6 D. T. McSwiggen, H. Liu, R. Tan, S. A. Puig, L. B. Akella, R. Berman, M. Bretan, H. Chen, X. Darzacq, K. Ford, R. Godbey, E. Gonzalez, A. Hanuka, A. Heckert, J. J. Ho, S. L. Johnson, R. Kelso, A. Klammer, R. Krishnamurthy, J. Li, K. Lin, B. Margolin, P. McNamara, L. Meyer, S. E. Pierce, A. Sule, C. Stashko, Y. Tang, D. J. Anderson and H. P. Beck, 2023, 2023.01.05.522916.
